# COVID-19 Incidence in Patients With Immunomediated Inflammatory Diseases: Influence of Immunosuppressant Treatments

**DOI:** 10.3389/fphar.2020.583260

**Published:** 2020-12-21

**Authors:** Natalia Soldevila-Domenech, Laura Tío, Jone Llorente-Onaindia, Elena Martín-García, Pau Nebot, Rafael de la Torre, Alba Gurt, Rafael Maldonado, Jordi Monfort, the Covidmar Study Group

**Affiliations:** ^1^ Integrative Pharmacology and Systems Neuroscience Research Group, Neurosciences Research Program, Hospital del Mar Medical Research Institute (IMIM), Barcelona, Spain; ^2^ Department of Experimental and Health Sciences, Universitat Pompeu Fabra (CEXS-UPF), Barcelona, Spain; ^3^ IMIM (Hospital Del Mar Medical Research Institute), PRBB, Barcelona, Spain; ^4^ Laboratory of Neuropharmacology, Department of Experimental and Health Sciences, Universitat Pompeu Fabra, PRBB, Barcelona, Spain; ^5^ Spanish Biomedical Research Centre in Physiopathology of Obesity and Nutrition (CIBERObn), Instituto de Salud Carlos III (ISCIII), Madrid, Spain; ^6^ CAP Vila Olímpica, Parc Sanitari Pere Virgili, Barcelona, Spain; ^7^ Rheumatology Service, Hospital del Mar, Barcelona, Spain

**Keywords:** biological therapy, tumor necrosis factor inhibitor, cross-sectional study, relative risk, disease modifying antirheumatic drugs (DMARDs), gender, glucocorticoids

## Abstract

The effect of immunosuppressant treatments on the incidence of coronavirus disease (COVID-19) remains largely unknown. We studied the association between the pre-exposure to disease-modifying antirheumatic drugs (DMARDs) that decrease immunological responses and the incidence of COVID-19 to explore the possible effects of these treatments in early manifestations of the disease. For this purpose, we performed a cross-sectional study including 2,494 patients with immunomediated inflammatory diseases (IMIDs) recruited at the outpatient Rheumatology, Dermatology and Gastroenterology services of Hospital del Mar. The primary outcome was the clinical diagnosis of COVID-19 performed by a physician at the hospital or at the primary care center, from the March 1–29, 2020. Multivariable Poisson regression models were fitted to estimate COVID-19 relative risk (RR) adjusted by comorbidities. We revealed that biological (RR = 0.46, CI 95% = 0.31–0.67) and synthetic (RR = 0.62, CI 95% = 0.43–0.91) DMARDs used in IMIDs diminished the incidence of COVID-19. Striking sex differences were revealed with anti-TNFα compounds (RR = 0.50, CI 95% = 0.33–0.75) with higher effects in women (RR = 0.33, CI 95% = 0.17–0.647). Treatment with low glucocorticoid doses also revealed sex differences decreasing the incidence of COVID-19 predominantly in women (RR = 0.72, CI 95% = 0.42–1.22). Our results report a decreased incidence of COVID-19 in patients receiving specific DMARDs with different immunodepressor mechanisms with striking sex differences. These results underline the interest of repurposing specific DMARDs for the possibility of minimizing the severity of disease progression in the early stages of COVID-19.

## Introduction

Since December 2019, cases of severe acute respiratory syndrome coronavirus 2 (SARS-CoV-2) infection leading to a novel disease called COVID-19 were initially identified in China. SARS-CoV-2 infection causes respiratory symptoms that range from mild forms of presentation to more serious ones that can risk patients’ lives, causing pneumonia, and damage to other organs, particularly the immune and blood system (Chen et al., 2020; Huang et al., 2020; Wang et al., 2020). This disease has rapidly expanded to multiple countries leading to a pandemic situation in March 2020 now affecting 7.360.239 individuals worldwide, with a global mortality of 416.201 deaths on June 11th. The situation has been dramatic in some European countries during the last months, such as Spain with 242.280 cases and 27.136 deaths (Dong et al., 2020). This official mortality numbers only reflect the casualties occurring in the hospitals, not in nursing homes or at home, and considering the low availability of accurate COVID-19 diagnostic tests, the current situation in Spain could unfortunately be worse. Furthermore, some patients are asymptomatic (Mizumoto et al., 2020; Nishiura et al., 2020) and the current prevalence reflects a possible underdiagnosis of the infection that has facilitated the disease expansion.

Immunomediated inflammatory diseases (IMIDs) are a group of unrelated and highly diverse conditions, such as rheumatoid arthritis and psoriasis, that share a common pathogenesis pathway, i.e., an immune dysregulation leading to an imbalance in inflammatory mediators. Treatments to relieve IMIDs are namely disease modifying antirheumatic drugs (DMARDs), subdivided into two main subgroups: synthetic (sDMARDs) and biological (bDMARDs). Both groups are aimed to decrease the hyperactivity of the immune system: bDMARDs are monoclonal antibodies presenting a much higher affinity and selectivity to their targets (mainly pro-inflammatory IL, and TNFα), while sDMARDs have a less selective immunosuppressant effect, except for Jak-inhibitors.

On the other side, evidence suggests that the hyperactivation of the immune response is of paramount relevance in COVID-19 progression. The accumulated knowledge about the pathophysiology of this disease reveals a crucial involvement of different molecules of the main inflammatory pathways, including interleukins 1, 6, and 8 (IL-1, IL-6, IL-8) and tumor necrosis factor alpha (TNFα). Drugs inhibiting some of these pathways have been used in the routine management of COVID-19, although results from clinical trials are still required to corroborate their effectiveness (Zhong et al., 2020). Clear examples are anti-IL-6 compounds for patients with severe forms of COVID-19 (Fu et al., 2020; Zhang et al., 2020; Zhou et al., 2020) and hydroxychloroquine, widely used and highly questioned (Adhanom Ghebreyesus, 2020; Mehra et al., 2020).

This similar physiopathology, as well as the mechanism of action of the drugs used for IMID management, has focused the attention on the study of patients suffering from IMID as a population of particular interest in the study of COVID-19 (Gianfrancesco et al., 2020a, Gianfrancesco et al., 2020b; Favalli et al., 2020; Michelena et al., 2020; Monti et al., 2020; Salvarani et al., 2020). Patients with an autoimmune disease might be at higher risk of developing severe infections, as these medications are immunosuppressants (Memoli et al., 2014). However, this assumption has not been confirmed for SARS-CoV-2 infection, as several studies describe that the COVID-19 incidence in IMID patients is similar to the general population (Memoli et al., 2014; Favalli et al., 2020; Michelena et al., 2020; Salvarani et al., 2020). Some studies have focused on the effect of IMID treatment on COVID-19 severity in terms of hospitalization and death. Thus, systemic glucocorticoid pretreatment was reported to represent a risk factor for severe COVID-19 (OR, 6.9; 95% CI, 2.3–20.5) in patients with inflammatory bowel disease, while anti-TNFα treatment presents no association (Brenner et al., 2020). On the other hand, the COVID-19 Global Rheumatology Alliance studied the demographic and clinical factors associated with COVID-19 hospitalization in rheumatic patients and found that a ≥10 mg/day glucocorticoid dose was associated with a higher odds of hospitalization (OR 2.05, 95% CI 1.06–3.96), whereas anti-TNFα present a decreased incidence or hospitalizations (OR 0.40, 95% CI 0.19–0.81). No association were observed neither with DMARDs nor antimalarial use (Gianfrancesco et al., 2020a; Gianfrancesco et al., 2020b). Similar results were reported in patients using immunomodulatory therapy, regardless of the underlying disease. Indeed, a trend to a higher incidence of hospitalization was observed with chronic glucocorticoid treatment <10 mg/day in these patients, while anti-TNFα use was associated with a reduced odd of hospitalization (Winthrop et al., 2020).

These studies generally use age-standardized rates, so they tackle the problem of comparing populations with different age structures. However, such populations may also differ considering their distribution of associated comorbidities and treatments for these comorbidities, which could influence the results. Furthermore, the majority of studies evaluated the effect of the treatment on developing severe symptoms, with limited data considering also mild to moderate symptoms. In that context, there is a need to study the COVID-19 incidence in IMID patients and the potential effect of immunosuppressants controlling for the influence of the different distribution of risk factors in order to evaluate the possibility of repurposing possible new drugs for COVID-19 therapy.

## Methods

### Study Design and Population

This is a cross-sectional study aimed to evaluate the effect of different DMARDs on the accumulated incidence of COVID-19 during March 2020 in patients with IMIDs living in Barcelona (Spain). The studied population was composed of 1) patients with IMIDs taking bDMARDs (exposed patients) and 2) patients with IMIDs or other musculoskeletal diseases that were not taking bDMARDs (unexposed patients). All patients had been visited at the outpatient Rheumatology, Dermatology and Gastroenterology services of Hospital del Mar (referral hospital from Barcelona) from September 2019 to March 2020.

The exclusion criteria were <18 years old, previous death not related with SARS-CoV-2 infection and patients tested negative for SARS-CoV-2 or without follow up at the primary care center during the studied period. The study was undertaken according to Good Clinical Practice guidelines and the Declaration of Helsinki. The research ethics review committee of Parc de Salut Mar approved the protocol (2020/9,246).

### Data Collection

A comprehensive review of the medical history of eligible patients was carried out using the registry of the Catalan national health system (eCAP). This register of the health system of Catalonia is a computerized medical history program that collects the health status of each of the patients and all entries to the public primary care system are recorded in this register. In turn, this database is fed by other information systems of the public network so that it contains continuously updated information on all consultations to hospitals, emergency services, pharmacy, death certifiers and any other relevant clinical information. The Hospital del Mar also has its own program of computerized medical record called IMASIS. Both database platforms were consulted for reviewing the medical histories and both are interconnected online. The immediate updating of the data in these platforms avoids any type of information loss. A clinical history revision of the included patients was performed from the 1st to March 29, 2020, focusing mainly at patient’s consulting disease, comorbidities and the treatments being currently followed by them (Supplementary Tables S1, S2). Briefly, diabetes, pulmonary disease, cardiovascular (CV) disease and chronic kidney disease were registered. In the case of arterial hypertension (AHT) and transplantation, they were only recorded if patients were receiving treatment with specific drugs for those comorbidities. Finally, cancer was recorded only if the patient had an active process or was following a treatment for a previous cancer, during the studied period.

The primary outcome was the clinical diagnosis of COVID-19 performed by a physician at the hospital or at the primary care center, from the 1st to March 29, 2020. In some patients, the diagnosis was complemented with a positive SARS-CoV-2 test, but in most of them it was based on clinical criteria following the Spanish health authorities’ recommendations: fever (defined as axillary temperature >37°C) together with shortness of breath and/or cough. If only fever was present, it was also considered as COVID-19 diagnosis if it appeared together with at least two of the following symptoms: anosmia, ageusia, rhinorrhea, diarrhea of one week of evolution, pharyngitis, odynophagia or arthromyalgia.

### Statistical Analysis

To evaluate the associations between different treatments and the diagnosis of COVID- 19, Poisson regression models with robust variance estimation were used to estimate relative risk (RR) and 95% confidence intervals (CI 95%). Models were adjusted by sex, age, diabetes, pulmonary disease, CV disease, chronic kidney disease, and active cancer or treatment. Model 1 aimed to estimate the association between treatments grouped by drug type 1) bDMARDs; 2) sDMARDs, 3) glucocorticoids, 4) chronic nonsteroidal anti-inflammatory drugs (NSAIDs) and 5) anti-hypertensive drugs. Then, associations between COVID-19 symptoms were estimated by each individual treatment (with >100 exposed patients; reference category = “unexposed”; Model 2). Finally, as anti-TNFα treatments were the major group of bDMARDs, the effect of each anti-TNFα drug was estimated separately in model 3. Model three also included the effect of anti-IL17 and anti-IL23 (−12), but anti-IL6 could not be analyzed as a separate group as there were not COVID-19 symptoms reported among individuals exposed to IL-6 antagonists. Interactions between different drug types were also tested (model 4). Finally, the main treatment indications for anti-TNFα, together with the studied comorbidities (sex, age, CV disease, diabetes, pulmonary disease, kidney disease and cancer) were used to create a matched dataset with propensity score matching based on the nearest neighbor method (Ho et al., 2011). Propensity score is the probability of exposure conditional upon confounders, estimated by logistic regression. Therefore, each treated individual was matched with an untreated individual whose propensity score was closest to that of the treated subject. Statistical analyses were performed using R (R Foundation for Statistical Computing, Vienna, Austria) version 3.5.2.

## Results

A total of 2,544 individuals were examined for eligibility and 2,494 fulfilled inclusion/exclusion criteria and were finally included in the analysis, 902 (36.2%) men and 1,592 (63.8%) women.

Tables 1, 2 show the description of the comorbidities and treatments followed by studied population. The mean age (SD) was 58.7 (15.7) and the most prevalent underlying pathologies were spondyloarthritis (32.6%), rheumatoid arthritis (21.6%) and osteoarthritis (25.1%). Almost half of individuals had at least one of the following comorbidities: hypertension (34%), diabetes (12.1%), pulmonary disease (14%), CV disease (11%), chronic kidney disease (5%), active cancer or treatment (3%) and post-transplant (0.3%). In terms of treatments, 45% of individuals were taking bDMARDs (59% in men and 36% in women), primarily anti- TNFα (30% in total; 42% in men and 24% in women). A third of the population were exposed to sDMARDs, being methotrexate, leflunomide and chloroquine/hydroxychloroquine the most prevalent ones (22%, 5% and 5%, respectively). Glucocorticoid consumption in women was twice that in men (26% vs 13%) but, in both cases, doses of glucocorticoids higher than 10 mg/day were unusual (<4%). NSAIDs and anti-hypertensive drugs were taken by the 20% and 27% of individuals, respectively. A 15.8% of the population (18.4% in women and 11.2% in men) did not take any of the registered treatments (Supplementary Table S3).

**TABLE 1:** Characteristics of the study population [N (%)].

Characteristic	All (N = 2,494)	Women (N = 1,592)	Men (N = 902)
Age [mean (SD)]	58.7 (15.7)	60.6 (15.5)	55.5 (15.6)
**Primary diagnosis**			
spondyloarthritis	812 (32.6%)	359 (22.6%)	453 (50.2%)
Rheumatoid arthritis	538 (21.6%)	424 (26.6%)	114 (12.6%)
Osteoarthritis	627 (25.1%)	480 (30.2%)	147 (16.3%)
Systemic autoimmune rheumatic diseases	165 (6.62%)	149 (9.36%)	16 (1.77%)
Vasculitis	59 (2.37%)	37 (2.32%)	22 (2.44%)
Other rheumatic diseases	38 (1.52%)	26 (1.63%)	12 (1.33%)
Juvenile arthritis	7 (0.28%)	4 (0.25%)	3 (0.33%)
Dermatological diseases	208 (8.34%)	82 (5.15%)	126 (14.0%)
Other	40 (1.60%)	31 (1.95%)	9 (1.00%)
**Coexisting conditions**			
Hypertension	858 (34.4%)	553 (34.7%)	305 (33.8%)
Diabetes	302 (12.1%)	174 (10.9%)	128 (14.2%)
Pulmonary disease	364 (14.6%)	241 (15.1%)	123 (13.6%)
CV Disease	290 (11.6%)	179 (11.2%)	111 (12.3%)
Chronic kidney disease	129 (5.17%)	76 (4.77%)	53 (5.88%)
Cancer or active treatment	70 (2.81%)	47 (2.95%)	23 (2.55%)
History of organ transplantation	8 (0.32%)	7 (0.44%)	1 (0.11%)
Any of these conditions	1,223 (49.0%)	797 (50.1%)	426 (47.2%)

**TABLE 2:** Characteristics of the study population [N (%)].

	All (N = 2,494)	Women (N = 1,592)	Men (N = 902)
**Treatments followed**			
Biologic DMARDs^1^	1,112 (44.6%)	579 (36.4%)	533 (59.1%)
Any TNFα antagonist	768 (30.8%)	388 (24.4%)	380 (42.1%)
Adalimumab	367 (14.7%)	163 (10.2%)	204 (22.6%)
Etanercept	183 (7.34%)	105 (6.60%)	78 (8.65%)
Infliximab	120 (4.81%)	60 (3.77%)	60 (6.65%)
Golimumab	65 (2.61%)	35 (2.20%)	30 (3.33%)
Certolizumab	33 (1.32%)	25 (1.57%)	8 (0.89%)
Any pro-inflammatory ILs antagonists	279 (11.2%)	136 (8.54%)	143 (15.9%)
IL-6 antagonists	52 (2.09%)	42 (2.64%)	10 (1.11%)
Tocilizumab	46 (1.84%)	37 (2.32%)	9 (1.00%)
Sarilumab	6 (0.24%)	5 (0.31%)	1 (0.11%)
IL-17 antagonists	69 (24.7%)	26 (19.1%)	43 (30.1%)
Brodalumab	2 (0.72%)	1 (0.74%)	1 (0.70%)
Secukinumab	51 (2.04%)	22 (1.38%)	29 (3.22%)
Ixekizumab	16 (5.73%)	3 (2.21%)	13 (9.09%)
IL-23 (12) antagonists	158 (56.6%)	68 (50.0%)	90 (62.9%)
Ustekinumab	155 (6.21%)	67 (4.21%)	88 (9.76%)
Guselkumab	3 (1.08%)	1 (0.74%)	2 (1.40%)
Any T lymphocyte antagonist	29 (1.16%)	22 (1.38%)	7 (0.78%)
Any B lymphocyte antagonist	42 (1.68%)	36 (2.26%)	6 (0.67%)
Vedolizumab	3 (0.12%)	2 (0.13%)	1 (0.11%)
Synthetic DMARDs^2^	850 (34.1%)	583 (36.6%)	267 (29.6%)
Methotrexate	538 (21.6%)	366 (23.0%)	172 (19.1%)
Leflunomide	116 (4.65%)	86 (5.40%)	30 (3.33%)
Chloroquine or hydroxychloroquine	115 (4.61%)	105 (6.60%)	10 (1.11%)
Azathioprine	80 (3.21%)	52 (3.27%)	28 (3.10%)
JAK inhibitors	41 (1.64%)	32 (2.01%)	9 (1.00%)
Apremilast	52 (2.09%)	20 (1.26%)	32 (3.55%)
Sulfasalazine	10 (0.40%)	7 (0.44%)	3 (0.33%)
Mycophenolate	19 (0.76%)	17 (1.07%)	2 (0.22%)
Tacrolimus	24 (0.96%)	17 (1.07%)	7 (0.78%)
Cyclosporine	3 (0.12%)	2 (0.13%)	1 (0.11%)
Dose of glucocorticoids	—	—	—
≤10 mg/d	441 (17.7%)	347 (21.8%)	94 (10.4%)
>10 mg/d	86 (3.45%)	62 (3.89%)	24 (2.66%)
Anti-hypertensive drugs^3^	684 (27.4%)	428 (26.9%)	256 (28.4%)
ACE inhibitors	397 (15.9%)	237 (14.9%)	160 (17.7%)
ARBs	293 (11.7%)	194 (12.2%)	99 (11.0%)
Chronic NSAIDs	498 (20.0%)	345 (21.7%)	153 (17.0%)

CV= cardiovascular. DMARDs = disease modifying anti-rheumatic drugs. JAK = Janus kinase. IL=interleukin. TNF=tumor necrosis factor. NSAIDs = non-steroid anti-inflammatory drugs. ACE = angiotensin-converting enzyme. ARBs = angiotensin II receptor blockers.

^1^Biologic DMARDs include TNF antagonists, pro-inflammatory ILs antagonists, vedolilzumab and T and B lymphocyte antagonists.

^2^Synthetic DMARDs include methotrexate, JAK inhibitors, sulfasalazine, mycophenolate, tacrolimus, azathioprine, cyclosporine, chloroquine or hydroxychloroquine and leflunomide and apremilast.

^3^Anti-hypertensive drugs include ACE inhibitors and ARBs.

In the cohort of individuals exposed to bDMARDs, the presence of the main comorbidities (hypertension, pulmonary disease and CV disease) was lower than in the cohort of individuals unexposed to bDMARDs. Also, their mean age (SD) was 52.2 (14.7) years, while in the cohort of unexposed to bDMARDs their mean age was 64 (15.4) years (see Supplementary Table 4 for further details).

The total number of patients with COVID-19 diagnosis was 156. As shown in Tables 3, 4, those presenting clinical diagnosis of COVID-19 had less spondyloarthritis, rheumatoid arthritis or dermatological diseases, and higher osteoarthritis. The proportion of diabetics in the group of individuals with COVID-19 was 20.5%, while in the group without symptoms was 11.5%. In the case of pulmonary disease, these percentages were 22.4% and 14.1%, respectively. The proportion of patients taking bDMARDS and sDMARDs was lower in the group with COVID-19 diagnosis. Among those with a clinical diagnosis of COVID-19, 32 were confirmed by a SARS-CoV-2 test and the remaining 124 had not been tested. There were 26 individuals (8 men and 18 women) hospitalized and there were 4 deaths due to COVID-19.

**TABLE 3:** Distribution of COVID-19 across categories of study variables.

	All		Women		Men	
	No symptoms (N = 2,338)	Symptoms (N = 156)	No symptoms (N = 1,484)	Symptoms (N = 108)	No symptoms (N = 854)	Symptoms (N = 48)
Age [mean (SD)]	58.5 (15.7)	62.1 (16.2)	60.3 (15.5)	64.8 (15.5)	55.5 (15.5)	56.0 (16.1)
**Primary diagnosis**						
spondyloarthritis	770 (32.9%)	42 (26.9%)	340 (22.9%)	19 (17.6%)	430 (50.4%)	23 (47.9%)
Rheumatoid arthritis	519 (22.2%)	19 (12.2%)	408 (27.5%)	16 (14.8%)	111 (13.0%)	3 (6.25%)
Osteoarthritis	563 (24.1%)	64 (41.0%)	424 (28.6%)	56 (51.9%)	139 (16.3%)	8 (16.7%)
Systemic autoimmune rheumatic diseases	159 (6.80%)	6 (3.85%)	145 (9.77%)	4 (3.70%)	14 (1.64%)	2 (4.17%)
Vasculitis	53 (2.27%)	6 (3.85%)	35 (2.36%)	2 (1.85%)	18 (2.11%)	4 (8.33%)
Other rheumatic diseases	26 (11.1%)	12 (7.69%)	22 (1.48%)	4 (3.70%)	9 (1.05%)	3 (6.25%)
Juvenile arthritis	7 (0.30%)	0 (0.00%)	4 (0.27%)	0 (0.00%)	3 (0.35%)	0 (0.00%)
Dermatological diseases	202 (8.64%)	6 (3.85%)	80 (5.39%)	2 (1.85%)	122 (14.3%)	4 (8.33%)
Other	31 (1.33%)	9 (5.77%)	26 (1.75%)	5 (4.63%)	8 (0.94%)	1 (2.08%)
**Coexisting conditions**						
Hypertension	788 (33.7%)	70 (44.9%)	505 (34.0%)	48 (44.4%)	283 (33.1%)	22 (45.8%)
Diabetes	270 (11.5%)	32 (20.5%)	152 (10.2%)	22 (20.4%)	118 (13.8%)	10 (20.8%)
Pulmonary disease	329 (14.1%)	35 (22.4%)	216 (14.6%)	25 (23.1%)	113 (13.2%)	10 (20.8%)
CV Disease	265 (11.3%)	25 (16.0%)	161 (10.8%)	18 (16.7%)	104 (12.2%)	7 (14.6%)
Chronic kidney disease	117 (5.00%)	12 (7.69%)	70 (4.72%)	6 (5.56%)	47 (5.50%)	6 (12.5%)
Cancer or activetreatment	64 (2.74%)	6 (3.85%)	43 (2.90%)	4 (3.70%)	21 (2.46%)	2 (4.17%)
History of organ transplantation	7 (0.30%)	1 (0.64%)	6 (0.40%)	1 (0.93%)	1 (0.12%)	0 (0.00%)
Any of these conditions	1,122 (48.0%)	101 (64.7%)	728 (49.1%)	69 (63.9%)	394 (46.1%)	32 (66.7%)

**TABLE 4:** Distribution of COVID-19 across categories of study variables.

	All		Women		Men	
	No symptoms (N = 2,338)	Symptoms (N = 156)	No symptoms (N = 1,484)	Symptoms (N = 108)	No symptoms (N = 854)	Symptoms (N = 48)
**Treatments followed**	—	—	—	—	—	—
Biologic DMARDs^1^	1,070 (45.8%)	42 (26.9%)	560 (37.7%)	19 (17.6%)	510 (59.7%)	23 (47.9%)
Any TNFα antagonist	739 (31.6%)	29 (18.6%)	378 (25.5%)	10 (9.26%)	361 (42.3%)	19 (39.6%)
adalimumab	353 (15.1%)	14 (8.97%)	159 (10.7%)	4 (3.70%)	194 (22.7%)	10 (20.8%)
Etanercept	178 (7.61%)	5 (3.21%)	104 (7.01%)	1 (0.93%)	74 (8.67%)	4 (8.33%)
Infliximab	114 (4.88%)	6 (3.85%)	57 (3.84%)	3 (2.78%)	57 (6.67%)	3 (6.25%)
golimumab	63 (2.69%)	2 (1.28%)	34 (2.29%)	1 (0.93%)	29 (3.40%)	1 (2.08%)
certolizumab	31 (1.33%)	2 (1.28%)	24 (1.62%)	1 (0.93%)	7 (0.82%)	1 (2.08%)
All pro-inflammatory ILs antagonists	269 (11.5%)	10 (6.41%)	130 (8.76%)	6 (5.56%)	139 (16.3%)	4 (8.33%)
IL-6 antagonists	52 (2.22%)	0 (0.00%)	42 (2.83%)	0 (0.00%)	10 (1.17%)	0 (0.00%)
IL-17 antagonists	68 (2.91%)	1 (0.64%)	26 (1.75%)	0 (0.00%)	42 (4.92%)	1 (2.08%)
IL-12/23 antagonists	149 (6.37%)	9 (5.77%)	62 (4.18%)	6 (5.56%)	87 (10.2%)	3 (6.25%)
T lymphocyte antagonists	27 (1.15%)	2 (1.28%)	20 (1.35%)	2 (1.85%)	7 (0.82%)	0 (0.00%)
B lymphocyte antagonists	42 (1.80%)	0 (0.00%)	36 (2.43%)	0 (0.00%)	6 (0.70%)	0 (0.00%)
vedolizumab	2 (0.09%)	1 (0.64%)	1 (0.07%)	1 (0.93%)	1 (0.12%)	0 (0.00%)
Synthetic DMARDs^2^	807 (34.5%)	43 (27.6%)	553 (37.3%)	30 (27.8%)	254 (29.7%)	13 (27.1%)
Methotrexate	510 (21.8%)	28 (17.9%)	348 (23.5%)	18 (16.7%)	162 (19.0%)	10 (20.8%)
Leflunomide	111 (4.75%)	5 (3.21%)	82 (5.53%)	4 (3.70%)	29 (3.40%)	1 (2.08%)
Apremilast	51 (2.18%)	1 (0.64%)	19 (1.28%)	1 (0.93%)	32 (3.75%)	0 (0.00%)
Chloroquine or hydroxychloroquine	108 (4.62%)	7 (4.49%)	99 (6.67%)	6 (5.56%)	9 (1.05%)	1 (2.08%)
JAK inhibitors	39 (1.67%)	2 (1.28%)	30 (2.02%)	2 (1.85%)	9 (1.05%)	0 (0.00%)
Sulfasalazine	9 (0.38%)	1 (0.64%)	7 (0.47%)	0 (0.00%)	2 (0.23%)	1 (2.08%)
Mycophenolate	18 (0.77%)	1 (0.64%)	16 (1.08%)	1 (0.93%)	2 (0.23%)	0 (0.00%)
Tacrolimus	22 (0.94%)	2 (1.28%)	15 (1.01%)	2 (1.85%)	7 (0.82%)	0 (0.00%)
Azathioprine	77 (3.29%)	3 (1.92%)	50 (3.37%)	2 (1.85%)	27 (3.16%)	1 (2.08%)
Cyclosporine	3 (0.13%)	0 (0.00%)	2 (0.13%)	0 (0.00%)	1 (0.12%)	0 (0.00%)
Glucocorticoids	—	—	—	—	—	—
≤10 mg/d	415 (17.8%)	26 (16.7%)	330 (22.2%)	17 (15.7%)	85 (9.95%)	9 (18.8%)
>10 mg/d	77 (3.29%)	9 (5.77%)	55 (3.71%)	7 (6.48%)	22 (2.58%)	2 (4.17%)
Anti-hypertensive drugs^3^	631 (27.0%)	53 (34.0%)	391 (26.3%)	37 (34.3%)	240 (28.1%)	16 (33.3%)
ACE inhibitors	375 (16.0%)	22 (14.1%)	221 (14.9%)	16 (14.8%)	154 (18.0%)	6 (12.5%)
ARBs	260 (11.1%)	33 (21.2%)	172 (11.6%)	22 (20.4%)	88 (10.3%)	11 (22.9%)
Chronic NSAIDs	461 (19.7%)	37 (23.7%)	320 (21.6%)	25 (23.1%)	141 (16.5%)	12 (25.0%)
**COVID-19 status**	—	—	—	—	—	—
SARS-CoV-2 test	—	—	—	—	—	—
Not tested	0 (0.00%)	122 (78.21%)	0 (0.00%)	87 (80.56%)	0 (0.00%)	35 (72.92%)
Positive	0 (0.00%)	34 (21.79%)	0 (0.00%)	21 (19.44%)	0 (0.00%)	13 (27.08%)
Hospitalization due to COVID-19	0 (0.00%)	26 (16.67%)	0 (0.00%)	18 (16.67%)	0 (0.00%)	8 (16.67%)
Deaths due to COVID-19	0 (0.00%)	4 (2.56%)	0 (0.00%)	2 (1.85%)	0 (0.00%)	2 (4.17%)

CV = cardiovascular. DMARDs = disease modifying anti-rheumatic drugs. JAK = Janus kinase. IL = interleukin. TNF = tumor necrosis factor. NSAIDs = non-steroid anti-inflammatory drugs. ACE = angiotensin-converting enzyme. ARBs = angiotensin II receptor blockers.

^1^Biologic DMARDs include anti-TNFα, pro-inflammatory ILs antagonists, vedolizumab and T and B lymphocyte antagonists.

^2^Synthetic DMARDs include methotrexate, JAK inhibitors, sulfasalazine, mycophenolate, tacrolimus, azathioprine, cyclosporine, chloroquine or hydroxychloroquine, leflunomide and apremilast.

^3^Anti‐hypertensive drugs include ACE inhibitors and ARBs.

**FIGURE 1:**
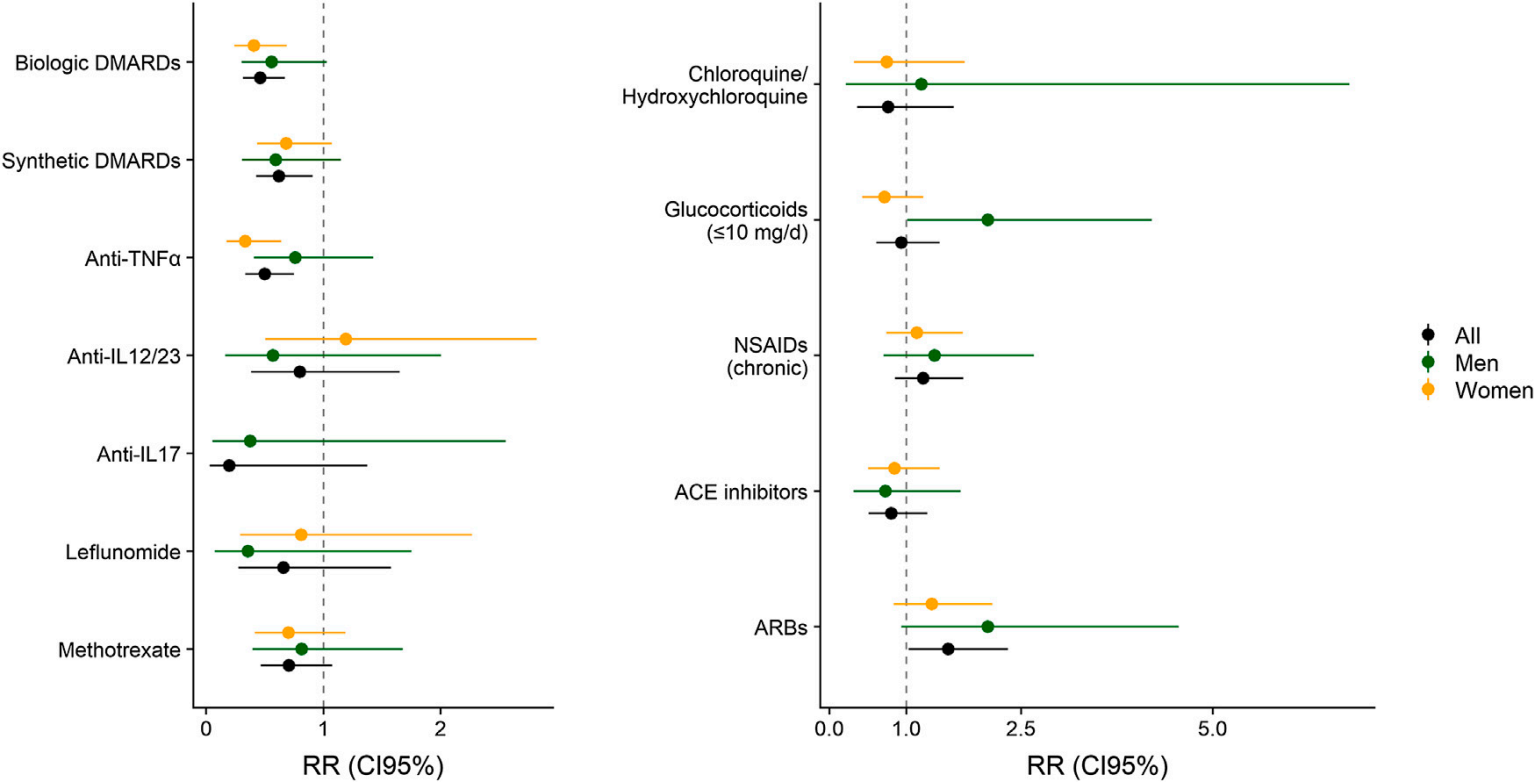
Relative Risk (RR) with 95% Confidence Interval (CI 95%) of COVID-19 according to the exposure to different treatments, adjusted by sex, age, CV disease, diabetes, pulmonary disease, chronic kidney disease and active cancer or treatments. The aggregated effect of biologic DMARDs, syntheticDMARDs, glucocorticoids and NSAIDs are obtained from Model 1. Estimates for Anti-TNFα, Anti-IL6/12/17/24 (anti-pro-inflammatory ILs), methotrexate, ACE inhibitors, ARBs and chloroquine/hydroxychloroquine are obtained from Model 2. Model 1 and 2 are represented in Tables 5, 6.

Adjusted associations between different exposure variables (clinical characteristics and treatments) and COVID-19 symptoms are shown in Tables 5, 6. This analysis allows to control the parameters that could be playing a role in the diagnosis of COVID- 19, such as sex, age, comorbidities, or treatments. Diabetes and pulmonary disease were associated with COVID−19 diagnosis, with overall RR_m1_ of 1.64 (CI 95% 1.09, 2.47) and 1.47 (CI 95% 1.02, 2.13). Regarding treatments, all bDMARDs presented an RR of 0.46 (CI 95% 0.31, 0.67) and all sDMARDs presented an RR of 0.62 (CI 95% 0.43, 0.91). Specifically, TNF-α antagonists presented RR of 0.50 (CI 95% 0.33, 0.75) in the whole population. This effect was even higher in women (RR = 0.33; CI 95% 0.17, 0.64), while in men the RR was 0.76 (CI 95% 0.41, 1.43), and given the risk difference ranging from 0.41 to 1.43, a substantial positive association was reasonably compatible with our data. All types of TNF-α antagonists (adalimumab, certolizumab, etanercept, golimumab and infliximab) showed RR estimates <1, although the differences were only statistically significant for adalimumab (RR = 0.53, CI 95% 0.31, 0.93) and etanercept (RR = 0.37, CI 95% 0.16, 0.88). The RR of anti-IL17 was 0.20 (CI 95% 0.03–1.38) and for anti-IL23 (12) was 0.80 (CI 95% 0.39, 1.65). Methotrexate and chloroquine/hydroxychloroquine presented a RR of 0.71 (CI 95% 0.46, 1.08) and 0.76 (CI 95% 0.36, 1.62), respectively. The RR of leflunomide was 0.66 (CI 95% 0.28, 1.58) in the whole population, with higher relative risk reduction in men (RR = 0.36; CI 95% 0.07, 1.75) than in women (RR = 0.81; CI 95% 0.29, 2.87). Glucocorticoids at doses of ≤10 mg/day also showed a relative risk reduction in women (RR = 0.72, CI 95% 0.42, 1.22). Figure 1 represents the adjusted RR for presenting COVID-19 symptoms according to the exposure to different treatments in men and women. The interactions between most prevalent combinations of treatments (bDMARDs + sDMARDs; bDMARDs + anti-hypertensive drugs; bDMARDs + chronic NSAIDs; sDMARDS + glucocorticoids) were included in Model 4 (Supplementary Table S6) and our results were most compatible with no important effects, except for the interaction between bDMARDs and cDMARDs (RR = 4.3; CI 95% 2.00, 9.25).

**TABLE 5:** Adjusted Relative Risk* (aRR) with 95% confidence intervals (CI 95%) of COVID-19 according to the presence of several comorbidities and treatments, stratified by sex.

	Model 1^A^- aRR (CI 95%)			Model 2^B^- aRR (CI 95%)			Model 3^C^- aRR (CI 95%)		
	All	Women	Men	All	Women	Men	All	Women	Men
Clinical characteristics	—	—	—	—	—	—	—	—	—
Women	1.12 (0.8, 1.57)	—	—	1.12 (0.8, 1.56)	—	—	1.12 (0.8, 1.57)	—	—
Age (years-old)	1 (0.99, 1.01)	1 (0.99, 1.02)	0.99 (0.97, 1.01)	1 (0.99, 1.01)	1 (0.99, 1.02)	0.99 (0.97, 1.01)	1 (0.99, 1.01)	1.01 (0.99, 1.02)	0.99 (0.97, 1.01)
CV Disease	1.12 (0.72, 1.74)	1.25 (0.75, 2.1)	0.85 (0.37, 1.99)	1.14 (0.74, 1.76)	1.23 (0.74, 2.05)	0.95 (0.42, 2.14)	1.13 (0.73, 1.74)	1.21 (0.73, 2.02)	0.95 (0.42, 2.13)
Diabetes	1.64 (1.09, 2.47)	1.74 (1.08, 2.8)	1.36 (0.62, 3)	1.61 (1.08, 2.42)	1.73 (1.08, 2.76)	1.26 (0.56, 2.82)	1.58 (1.05, 2.37)	1.67 (1.04, 2.68)	1.27 (0.56, 2.85)
Pulmonary disease	1.47 (1.02, 2.13)	1.5 (0.97, 2.32)	1.33 (0.66, 2.72)	1.42 (0.98, 2.05)	1.47 (0.94, 2.27)	1.28 (0.62, 2.62)	1.44 (0.99, 2.08)	1.48 (0.95, 2.3)	1.25 (0.61, 2.57)
Kidney disease	1.21 (0.65, 2.25)	0.87 (0.38, 1.99)	2.07 (0.76, 5.68)	1.19 (0.64, 2.21)	0.9 (0.39, 2.06)	1.83 (0.65, 5.13)	1.2 (0.65, 2.23)	0.89 (0.4, 2.01)	1.84 (0.67, 5.06)
Active cancer or treatment	1.15 (0.52, 2.58)	1.05 (0.38, 2.88)	1.43 (0.39, 5.24)	1.14 (0.51, 2.57)	1.05 (0.38, 2.88)	1.44 (0.36, 5.72)	1.13 (0.5, 2.55)	1.06 (0.39, 2.87)	1.44 (0.36, 5.75)

**TABLE 6:** Adjusted Relative Risk* (aRR) with 95% confidence intervals (CI 95%) of COVID-19 according to the presence of several.

	Model 1^A^- aRR (CI 95%)			Model 2^B^- aRR (CI 95%)			Model 3^C^- aRR (CI 95%)		
	All	Women	Men	All	Women	Men	All	Women	Men
**Treatments followed**	—	—	—	—	—	—	—	—	—
Biologic DMARDs^1^	0.46 (0.31, 0.67)	0.41 (0.24, 0.69)	0.56 (0.3, 1.03)	—	—	—	—	—	—
TNFα antagonists	—	—	—	0.50 (0.33, 0.75)	0.33 (0.17, 0.64)	0.76 (0.41, 1.43)	—	—	—
Adalimumab	—	—	—	—	—	—	0.53 (0.31, 0.92)	0.32 (0.12, 0.86)	0.81 (0.38, 1.75)
Certolizumab	—	—	—	—	—	—	0.86 (0.22, 3.34)	0.58 (0.08, 4.01)	1.68 (0.34, 8.2)
Etanercept	—	—	—	—	—	—	0.37 (0.16, 0.88)	0.13 (0.02, 0.97)	0.71 (0.27, 1.9)
Golimumab	—	—	—	—	—	—	0.46 (0.12, 1.81)	0.42 (0.06, 2.94)	0.56 (0.07, 4.28)
Infliximab	—	—	—	—	—	—	0.71 (0.31, 1.64)	0.7 (0.22, 2.23)	0.81 (0.24, 2.71)
Anti- pro-inflammatory ILs (IL6/12/17/23)	—	—	—	0.47 (0.24, 0.92)	0.57 (0.24, 1.34)	0.44 (0.15, 1.27)	—	—	—
Anti-IL17	—	—	—	—	—	—	0.2 (0.03, 1.38)	NA	0.37 (0.05, 2.56)
Anti-IL23 (12)	—	—	—	—	—	—	0.8 (0.39, 1.65)	1.19 (0.5, 2.82)	0.57 (0.16, 2)
Synthetic DMARDs^2^	0.62 (0.43, 0.91)	0.68 (0.43, 1.07)	0.59 (0.31, 1.15)	—	—	—	—	—	—
Methotrexate	—	—	—	0.71 (0.46, 1.08)	0.7 (0.42, 1.19)	0.81 (0.4, 1.68)	0.74 (0.48, 1.12)	0.74 (0.44, 1.24)	0.84 (0.41, 1.72)
Leflunomide	—	—	—	0.66 (0.28, 1.58)	0.81 (0.29, 2.27)	0.36 (0.07, 1.75)	0.66 (0.27, 1.57)	0.8 (0.28, 2.23)	0.36 (0.07, 1.79)
Chloroquine/Hydroxychloroquine	—	—	—	0.76 (0.36, 1.62)	0.75 (0.32, 1.76)	1.2 (0.21, 6.79)	0.81 (0.38, 1.71)	0.79 (0.34, 1.86)	1.27 (0.23, 7.16)
Glucocorticoids	—	—	—	—	—	—	—	—	—
≤10 mg/day	0.94 (0.61, 1.43)	0.72 (0.42, 1.22)	2.06 (1.01, 4.21)	0.87 (0.57, 1.33)	0.67 (0.4, 1.12)	2.05 (0.97, 4.3)	0.84 (0.55, 1.29)	0.65 (0.39, 1.1)	1.94 (0.93, 4.04)
>10 mg/day	1.76 (0.90, 3.45)	1.62 (0.75, 3.52)	2.20 (0.53, 9.24)	1.69 (0.87, 3.27)	1.61 (0.75, 3.43)	1.78 (0.43, 7.39)	1.7 (0.88, 3.3)	1.71 (0.8, 3.68)	1.78 (0.43, 7.34)
Anti-hypertensive^3^	1.08 (0.76, 1.52)	1.04 (0.7, 1.54)	1.11 (0.56, 2.21)	—	—	—	—	—	—
ACE inhibitors	—	—	—	0.81 (0.51, 1.28)	0.85 (0.50, 1.44)	0.73 (0.31, 1.71)	0.8 (0.51, 1.27)	0.84 (0.5, 1.43)	0.72 (0.3, 1.68)
ARBs	—	—	—	1.55 (1.03, 2.33)	1.33 (0.84, 2.13)	2.07 (0.94, 4.56)	1.59 (1.06, 2.39)	1.36 (0.85, 2.18)	2.11 (0.95, 4.66)
Chronic NSAIDs	1.22 (0.85, 1.75)	1.14 (0.74, 1.74)	1.37 (0.71, 2.67)	1.2 (0.84, 1.71)	1.12 (0.73, 1.7)	1.29 (0.67, 2.49)	1.21 (0.85, 1.72)	1.13 (0.74, 1.72)	1.31 (0.67, 2.58)

^*^Reference categories for clinical characteristics are individuals without that comorbidity. Reference categories for treatments are unexposed individuals.

^A^Model 1 contains the following explanatory or exposure variables: sex, age, CV disease, pulmonary disease, kidney disease, active cancer or treatment, biologic DMARDs, synthetic DMARDs, glucocorticoids, anti-hypertensive drugs and chronic NSAIDs.

^B^Model 2 contains the following explanatory or exposure variables: sex, age, CV disease, pulmonary disease, kidney disease, active cancer or treatment, TNFα antagonists, IL-6/12/17/23 antagonists, methotrexate, leflunomide, chloroquine/hydroxychloroquine, glucocorticoids, ACE inhibitors, ARBs and chronic NSAIDs.

^C^Model 3 contains the following explanatory or exposure variables: sex, age, CV disease, pulmonary disease, kidney disease, active cancer or treatment, adalimumab, certolizumab, Etanercept, golimumab, infliximab, anti- IL17, anti-IL12/23, methotrexate, leflunomide, chloroquine/hydroxychloroquine, glucocorticoids, ACE inhibitors, ARBs and chronic NSAIDs.

CV = cardiovascular. DMARDs = disease modifying anti-rheumatic drugs. JAK = Janus kinase. IL = interleukin. TNF = tumor necrosis factor. NSAIDs = non-steroid anti-inflammatory drugs. ACE = angiotensin-converting enzyme. ARBs = angiotensin II receptor blockers. N = number of observations or exposed individuals.

^1^Biologic DMARDs include TNF antagonists, pro-inflammatory ILs antagonists, vedolizumab and T and B lymphocyte antagonists.

^2^Synthetic DMARDs include methotrexate, JAK inhibitors, sulfasalazine, mycophenolate, tacrolimus, azathioprine, cyclosporine, chloroquine or hydroxychloroquine, leflunomide and apremilast.

^3^Anti-hypertensive drugs include ACE inhibitors and ARBs.

Finally, the crude RR using propensity score matching for the exposure to anti-TNFα was 0.80 (CI 95% 0.50, 1.30) and the adjusted RR (by anti-pro-inflammatory ILs, methotrexate, leflunomide, chloroquine/hydroxychloroquine, glucocorticoids, ACE inhibitors, ARBs, NSAIDs) was 0.69 (CI 95% 0.38, 1.23). A description of the matched dataset is included in Supplementary Table S7.

## Discussion

Our cross-sectional study reveals that the DMARDs treatments commonly used in IMIDs are not associated with an increase in COVID-19 incidence. All the treatments analyzed in our study were not discontinued in our cohorts of patients following the previous recommendations (Gianfrancesco et al., 2020a, Gianfrancesco et al., 2020b; Haberman et al., 2020; Michelena et al., 2020). It is important to underline that the primary outcome of our study was the manifestation of mild symptoms of COVID-19. Therefore, our results do not provide relevant information about the possible influence of these treatments in the severity of COVID-19, taking into account the low incidence of severe symptoms, hospitalizations and deaths in our cohort or early symptomatic patients. However, several studies have already reported that some IMID treatments have a protective effect on the incidence of developing severe symptoms, probably blocking the hyperactivation of the immune response occurring in the COVID-19 progression (Gianfrancesco et al., 2020a, Gianfrancesco et al., 2020b; Winthrop et al., 2020). Interestingly, in our study bDMARDs (RR = 0.46; CI 95% 0.31, 0.67) and sDMARDs (RR = 0.62; CI 95% 0.43, 0.91) treatment diminished the incidence of COVID-19, in agreement with previous preliminary observations (Haberman et al., 2020; Michelena et al., 2020). Therefore these treatments are also playing a role in the capacity to be infected by SARS-CoV-2 and/or in presenting mild symptoms of COVID-19. At these early stages of the disease, the two co-morbidities that significantly enhanced COVID-19 diagnosis in these group of patients were diabetes (RR = 1.64; CI 95% 1.09, 2.47) and pulmonary disease (RR = 1.47; CI 95% 1.02, 2.13). A large number of patients treated with bDMARDs (1,153) and sDMARDs (850 patients, 283 also receiving bDMARDs) has been included in our cohort. Therefore, the global decrease in the incidence of COVID-19 on patients treated with DMARDs has influenced the RR estimated for compounds that are supposed to not modify COVID-19 progression.

The protective effects of the anti-TNFα treatment on the incidence of COVID-19 symptoms reported in our study (RR = 0.50; CI 95% 0.33, 0.75) fully agree with the comments recently published about the urgent need of clinical trials of anti-TNFα therapy for COVID-19 (Feldmann et al., 2020; Robinson et al., 2020). Indeed, previous studies have reported that rheumatic patients treated with anti-TNFα present a decreased incidence of hospitalizations (OR 0.40, 95% CI 0.19–0.81) (Gianfrancesco et al., 2020a; Gianfrancesco et al., 2020b) and this protective effect was also observed in anti-TNFα treated patients regardless of the underlying disease (Winthrop et al., 2020). Our findings corroborate these protective effects considering the incidence of mild symptoms as the primary output of the study. Therefore, anti-TNFα treatment may have protective effects in the incidence of COVID-19 symptoms (our study), but also in the progression to severe manifestations of this disease (Gianfrancesco et al., 2020a, Gianfrancesco et al., 2020b; Winthrop et al., 2020). All together, these studies underlie the urgent need of clinical trials to obtain additional evidences of the possible efficacy of anti-TNFα treatment on COVID-19 (Robinson et al., 2020). Anti-TNFα therapy has been proposed to be initiated as early as is practicable in hospitalized patients with COVID-19 in order to obtain the possible optimal beneficial effects (Feldmann et al., 2020).

Although the studied population was not sex-balanced (1,592 women vs. 902 men) our analyses stratified by sex also revealed potential sex differences in the effects of several immunomodulatory compounds on the incidence of COVID-19 mild symptoms. Indeed, anti-TNFα compounds showed a decreased COVID-19 incidence that was higher in women (RR = 0.33; CI 95% 0.17, 0.64) than in men (RR = 0.76; CI 95% 0.41, 1.43). Although a possible sex influence in the therapeutic effects of anti-TNFα compounds is controversial, a positive female sex influence was already reported in the prognosis of ulcerative colitis in patients treated with infliximab, an anti-TNFα monoclonal antibody (Nasuno et al., 2017). Sex differences were also revealed in our study in the effects of glucocorticoids. Taken into account the high variability of the doses of glucocorticoids used in these patients (Ruiz-Irastorza et al., 2012) and the differential effects depending on dose exposure (Meng et al., 2020), we have stratified glucocorticoid treatment in low (≤10 mg of prednisone or equivalent) and high doses (>10 mg). Low glucocorticoids doses decreased COVID-19 incidence in women (RR = 0.72; CI 95% 0.42, 1.22), whereas high doses seemed to produce the opposite effect (RR = 1.62; CI 95% 0.75, 3.52).

Considering the high availability and the safety profile of low doses of glucocorticoids, this result could be of potential interest to further evaluate the possible benefits of using such low doses in women in early periods of SARS-CoV-2 infection to prevent progression of the disease. In contrast, the effects of leflunomide treatment were more clearly revealed in men (RR = 0.36; CI 95% 0.07, 1.75) than in women (RR = 0.81; CI 95% 0.29, 2.27). In line with our results, a significant clinical effect of leflunomide, particularly in male rheumatoid arthritis patients, has been reported. This could be explained by the synergistic effect of testosterone and leflunomide on proinflammatory cytokine production (Cutolo et al., 2009).

In the case of pre-exposure to anti-IL-17 and anti-IL-23, we observed a reduced COVID- 19 incidence (RR = 0.2; CI 95% 0.03, 1.38; and RR = 0.8; CI 95% 0.39, 1.65, respectively). It has been reported that patients infected with SARS-CoV-2 presented elevated IL-17 serum levels (Liu et al., 2020), which are significantly correlated with disease severity (Pacha et al., 2020; Schett et al., 2020). Due to its high capacity to promote the production of a vast amount of pro-inflammatory cytokines and chemokines, some authors have described that IL−17 and, therefore, the T helper 17 (TH^17^) response, play a role in COVID-19 hyperinflammation (Wu and Yang, 2020). Taking into account that IL-23 participates in stabilization of TH^17^ cells, our results support the idea (Liu et al., 2020) that targeting this axis could have a positive effect in controlling the cytokine storm.

However, our cohort includes limited number of patients treated with two important groups of immunomodulatory compounds, IL-6 (52 patients) and B lymphocyte antagonists (42 patients). Interestingly, none of these 94 patients showed COVID-19 symptoms, which agrees with the reported efficacy of the IL-6 antagonists tocilizumab (Xu et al., 2020) and sarilumab (unpublished observations) in COVID-19 treatment. The three families of monoclonal antibodies approved to treat rheumatoid arthritis are directed against IL-6, B lymphocyte surface protein CD20 and TNFα, three targets of potential interest for further investigation in COVID-19 treatment. IL-6, TNFα and B lymphocytes have been reported to play a crucial role in the inflammatory cascade taking place days before the manifestation of the most severe forms of SARS-CoV-2 infection (Zhou et al., 2020), as well as in the physiopathological processes leading to rheumatoid arthritis (Ceribelli et al., 2020).

In spite of the decrease incidence of COVID-19 with bDMARDs and sDMARDs treatments, those patients receiving a combination of both groups of compounds (n = 298) show enhanced incidence of COVID-19 (RR = 4.3; CI 95% 2.00, 9.25). The strong immunosuppression that should result by the combination of these treatments and the severity of the diseases targeted by these drug combinations may explain this paradoxical effect. Indeed, previous studies have reported that more patients experienced infectious adverse events when increasing doses of synthetic DMARDs were combined with anti-TNFα compounds (Burmester et al., 2015; Honkila et al., 2019). In addition, the main reason for combining both treatments is related to the lack of efficacy in these particular patients (Van Vollenhoven et al., 2012), which could also have influenced our results.

Some limitations of this study must be addressed. The indications for each treatment not only depend on the underlying pathology, but also on the specific clinical manifestations of each patient, and some of the indications are risk factors of COVID-19 (Sawalha et al., 2020). Given the heterogeneity of the studied treatments and underlying pathologies, it is difficult to analyze all the factors that could cause confounding by indication. However, RR estimates of COVID-19 diagnosis after propensity score matching with some of the covariates that predict receiving anti-TNFα were not substantially different than RR estimates in the unmatched sample (Supplementary Table S7). The slightly different RRs found with this treatment matching the above mention covariates suggest that some of these IMID may represent an increased risk for COVID-19. Indeed, these particular comorbidities have been reported to increase COVID-19 susceptibility and severity (Sawalha et al., 2020). Furthermore, patients receiving these immunomodulatory treatments have an enhanced propensity to bacterial infection (Chiu and Chen, 2020) that could eventually provide manifestations similar to COVID-19. In spite of this possible bias that would impair the results obtained with these treatments, we have obtained promising RRs with these compounds that suggest significant protective effects on COVID-19. Furthermore, our study was focused on the early stages of COVID-19 pandemic in Spain, and the number of confirmed SARS-CoV-2 testing in our setting was limited due to the scarcity of COVID-19 tests in Spain that, for ethical reasons, were mainly reserved to patients showing more severe disease symptoms. Therefore, clinical COVID-19 diagnosis was used as the primary outcome. Consequently, the effect of the treatment could play a role both in the risk to acquire the infection, and/or the risk of being asymptomatic. Finally, it is also important to underline that the clinical symptoms of COVID-19 were recorded from 14 days before the COVID-19 alarm was announced in Spain (March 16th) when patients could be supposed to protect themselves more if they are at risk. Therefore, this potential self-protection would not represent any important bias for the interpretation of our results considering the time schedule of our symptoms recording.

In summary, all these results suggest that bDMARDs and sDMARDs should be continued for IMIDs treatment in COVID-19 patients. The decreased incidence of COVID-19 in patients treated with anti-TNFα and anti-proinflammatory ILs compounds underline the potential interest of these medications for further studies to open novel possible therapeutic strategies to avoid serious COVID-19 manifestations.

## Data Availability Statement

The raw data supporting the conclusions of this article will be made available by the authors, without undue reservation, to any qualified researcher.

## Ethics Statement

The Committee of Parc de Salut Mar approved the protocol (2020/9246); IMIM (Hospital del Mar Medical Research Institute), PRBB, c/ Dr. Aiguader, 88, 08003 Barcelona. The ethics committee waived the requirement of written informed consent for participation.

## Covidmar Study Group Members

The Covidmar Study Group members are: Hospital del Mar, Barcelona: Selene Labrada, Miguel Meja-Torres (Rheumatology Service) and Irene Carrin-Barber, Carolina Prez-Garca, Fabiola Ojeda, Tarek Carlos Salman-Monte, Josep Blanch-Rubi (Rheumatology Service and IMIM-Hospital del Mar Medical Research Institute) collected data and provided care for study patients; IMIM-Hospital del Mar Medical Research Institute: Luciano Polino, Laura Triginer, Anna Ribes (Cell Research on Inflammation and Cartillage Research Group, Inflammatory and Cardiovascular Processes Program) collected data; Maria-Victria Puig (Integrative Pharmacology and Systems Neuroscience Research Group, Neurosciences Research Program and IMIM-Hospital del Mar Medical Research Institute); contributed to analysis design; Parc Sanitari Pere Virgili, Barcelona: Maria Teresa Mart Vila, Maria Luisa Perez Miras (CAP Vila Olmpica) collected data; Universitat Pompeu Fabra, Barcelona: Beltrn lvarez-Prez, Araceli Bergad-Martnez, Pablo Calv Alba Calvet-Pavn, Mireia Carcol, Laura Domingo-Rodrguez, Alejandra Escudero-Lara, Lorena Galera-Lpez, Jolita Jančytė, Marta Linares-Lpez, Sara Martnez-Torres, Antonio Ortega-lvaro, Andrs Ozaita, Sheila Piedra-Barrull, Dulce Real-Muoz, Maria Sanchis-Oll, Clara Seira Oriach, Miquel-ngel Serra, Anna Vzquez-Oliver (Laboratory of Neuropharmacology, Department of Experimental and Health Sciences and IMIM- Hospital del Mar Medical Research Institute) collected data.

## Author Contributions

NS-D participated in selection of statistical tests/analyses, performed the statistical analyses, computations and related computer work, and participated in writing the manuscript. LT was involved in conceptualizing the research idea, setting-up the research design, making the primary interpretation of the statistical analyses and participated in writing the manuscript. JL-O was involved in conceptualizing the research idea, setting-up the research design, making the primary interpretation of the statistical analyses and participated in writing the manuscript. EM-G contributed to the statistical analyses and revised the manuscript. PN contributed to the statistical analyses and revised the manuscript. RT contributed to the statistical analyses and revised the manuscript. AG was responsible for patient cohort data collection. RM was involved in conceptualizing the research idea, creating the research design, making the final interpretation of the statistical analysis, and writing the first draft and revision of the manuscript. JM was involved in conceptualizing the research idea, creating the research design, making the final interpretation of the statistical analysis, and writing the first draft and revision of the manuscript. Covidmar Study Group participated in collecting data and provided care for study patients.

## Funding

“Ministerio de Ciencia, Innovación y Universidades” (#AEI-SAF2017-84060-R FEDER to RM, #DPI2016-80283-C2-2-R), “Ministerio de Sanidad, Servicios Sociales e Igualdad” (#RD16/0017/0020 and #PNSD-2017I068 to RM, #PI18/00059 to TCS-M) and “Generalitat de Catalunya” (#2017-SGR-669 and #ICREA-Acadèmia 2015 to RM, #2017-SGR-138 to RdlT). NSD is recipient of predoctoral fellowship #2019-DI-47 from the DIUE-AGAUR of the “Generalitat de Catalunya.” The submitted work was supported by the Hospital del Mar.

## Conflict of Interest

AG has received research grants or consulting fees from Astrazeneca and Bioiberica S.A.U., RM has received research grants or consulting fees from Aelis, Almirall, Boehringer Ingelheim, BrainCo, Esteve, Ferrer, GlaxoSmithKline, Grünenthal, GW Pharmaceuticals, Janus, Lundbeck, Pharmaleads, Phytoplant, Rhodes, Sanofi, Spherium, Union de Pharmacologie Scientifique Appliquée, Upjohn, and Uriach; JM has received grants or consulting fees from Procare Health Iberia S.L, Esteve, Labhra, Bioibérica S.A.U, Grunenthal Pharma S.A, Pfizer, OPKO Heath Spain S.L.U and Roche Pharma S.A.

The remaining authors declare that the research was conducted in the absence of any commercial or financial relationships that could be construed as a potential conflict of interest.
