# Supplementary material for: COVID-19 Incidence in Patients With Immunomediated Inflammatory Diseases: Influence of Immunosuppressant Treatments
Source: Front Pharmacol. 2020 Dec 21;11:583260. doi: 10.3389/fphar.2020.583260 (PMC7845571; doi:10.3389/fphar.2020.583260)

Supplementary Material

**Supplementary Methods**

**Supplementary Table 1. Definition of the comorbidities registered**

| **Comorbidities** | **Definition** |
| --- | --- |
| AHT | Diagnosis of arterial hypertension in actual treatment with antihypertensive drugs. |
| Diabetes | Diagnosis of diabetes, treated with diet, oral drugs or insulin administration. |
| Pulmonary disease | Any disease affecting respiratory system functionality and treated with any of the related drugs. Patients suffering from asthma, Chronic obstructive pulmonary disease (COPD), Pulmonary fibrosis, Obstructive sleep apnea syndrome (OSAS) and interstitial lung disease were specifically registered. |
| CV disease | Diagnosis of cardiovascular disease |
| Chronic kidney disease | Chronic kidney disease diagnosis, in any of the stages. |
| Active cancer or treatment | Patients in a cancer process or in treatment for a previous cancer, along the period studied (1st to the 29th of March) |
| Tissue or organ transplantation | Any transplantation throughout the patient's life, in actual treatment. |

**Supplementary Table 2. List of types of treatments registered and drugs belonging to each type**

| **Type of treatment** | **Drugs included** | |
| --- | --- | --- |
| Biologic DMARDs | Anti-pro- inflammatory ILs | IL-6 antagonists: Sarilumab, Tocilizumab |
|  |  | IL-17 antagonists: Secukinumab, Ixekizumab, Brodalumab |
|  |  | IL-23(12) antagonists: Ustekinumab, Guselkumab, |
|  | Anti-TNFα: Etanercept, Infliximab, Adalimumab, Golimumab, Certolizumab | |
|  | T lymphocyte antagonists: Abatacept | |
|  | B lymphocyte antagonists: Rituximab, Belimumab. | |
|  | Vedolizumab | |
| Synthetic DMARDs | Methotrexate | |
|  | Sulfasalazine | |
|  | Mycophenolate | |
|  | Tacrolimus | |
|  | Azathioprine | |
|  | Cyclosporine | |
|  | Chloroquine or Hydroxychloroquine | |
|  | Leflunomide | |
|  | Apremilast | |
|  | JAK inhibitors: Tofacitinib, Baricitinib, Upadacitinib | |
| Corticoids | Any type of oral corticoid (all converted to prednisone dose) | |
| Antihypertensive drugs | Angiotensin-converting enzyme (Enalapril, Ramipril, Lisinopril, …) | |
|  | Angiotensin II Receptor Blockers (Losartan, Valsartan, Candesartan…) | |

**Supplementary Results**

**Supplementary Table 3. 10 most prevalent treatment profiles of the study population**

| **Biologic DMARDs^1^** | **Synthetic DMARDs^2^** | **Corticoids** | **Chronic NSAIDs** | **Anti- hypertensive^3^** | **All** | | **Women** | | **Men** | |  |
| --- | --- | --- | --- | --- | --- | --- | --- | --- | --- | --- | --- |
|  |  |  |  |  | N | % | N | % | N | % |  |
| X | - | - | - | - | 518 | 20.77 | 234 | 14.70 | 284 | 31.49 |  |
| - | - | - | - | - | 394 | 15.80 | 293 | 18.40 | 101 | 11.20 |  |
| - | - | - | - | X | 203 | 8.14 | 146 | 9.17 | 57 | 6.32 |  |
| - | X | - | - | - | 182 | 7.30 | 113 | 7.10 | 69 | 7.65 |  |
| - | X | X | - | - | 128 | 5.13 | 108 | 6.78 | 20 | 2.22 |  |
| X | X | - | - | - | 117 | 4.69 | 73 | 4.59 | 44 | 4.88 |  |
| X | - | - | - | X | 115 | 4.61 | 43 | 2.70 | 72 | 7.98 |  |
| - | - | - | X | - | 80 | 3.21 | 62 | 3.89 | 18 | 2.00 |  |
| - | X | - | - | X | 59 | 2.37 | 36 | 2.26 | 23 | 2.55 |  |
| X | - | - | X | - | 59 | 2.37 | 29 | 1.82 | 30 | 3.33 |  |
| CV= cardiovascular. DMARDs= disease modifying antirheumatic drugs. JAK=Janus kinase. IL=interleukin. TNF=tumor necrosis factor. NSAIDs= non-steroid anti-inflammatory drugs. ACE= angiotensin-converting enzyme. ARBs= angiotensin II receptor blockers.  1Biologic DMARDs include Anti-TNF-α, IL-6/12/17/23 antagonists, vedolizumab and T and B lymphocyte antagonists.  2Synthetic DMARDs include methotrexate, JAK inhibitors, sulfasalazine, mycophenolate, tacrolimus, azathioprine, cyclosporine, chloroquine or hydroxychloroquine, leflunomide and apremilast.  3Anti-hypertensive drugs include ACE inhibitors and ARBs. | | | | | | | | | | | |

**Supplementary Table 4. Characteristics of the cohort of individuals exposed to bDMARDs and the cohort of individuals unexposed to bDMARDs.**

|  | **Exposed to bDMARDs (N=1112)** | **Unexposed to bDMARDs (N=1382)** |
| --- | --- | --- |
| Women | 579 (52.1%) | 1013 (73.3%) |
| Age [mean (SD)] | 52.2 (14.7) | 64.0 (14.4) |
| **Primary diagnosis** |  |  |
| Spondylarthritis | 630 (56.7%) | 182 (13.2%) |
| Rheumatoid Arthritis | 281 (25.3%) | 257 (18.6%) |
| Osteoarthritis | 0 (0.00%) | 627 (45.4%) |
| Systemic autoimmune rheumatic diseases | 16 (1.44%) | 149 (10.8%) |
| Vasculitis | 10 (0.90%) | 49 (3.55%) |
| Other rheumatic diseases | 2 (0.18%) | 21 (1.52%) |
| Juvenile Arthritis | 6 (0.54%) | 1 (0.07%) |
| Dermatological diseases | 165 (14.8%) | 43 (3.11%) |
| Other | 2 (0.18%) | 53 (3.84%) |
| **Coexisting conditions** |  |  |
| Hypertension | 300 (27.0%) | 558 (40.4%) |
| Diabetes | 120 (10.8%) | 182 (13.2%) |
| Pulmonary disease | 125 (11.2%) | 239 (17.3%) |
| CV disease | 94 (8.45%) | 196 (14.2%) |
| Chronic kidney disease | 46 (4.14%) | 83 (6.01%) |
| Cancer or active treatment | 11 (0.99%) | 59 (4.27%) |
| History of organ transplantation | 2 (0.18%) | 6 (0.43%) |
| Any of these conditions | 440 (39.6%) | 783 (56.7%) |
| Synthetic DMARDs | 283 (25.4%) | 567 (41.0%) |
| Methotrexate | 156 (14.0%) | 382 (27.6%) |
| Leflunomide | 48 (4.32%) | 68 (4.92%) |
| Apremilast | 7 (0.63%) | 45 (3.26%) |
| Chloroquine or Hydroxychloroquine | 13 (1.17%) | 102 (7.38%) |
| JAK inhibitors | 0 (0.00%) | 41 (2.97%) |
| Sulfasalazine | 2 (0.18%) | 8 (0.58%) |
| Mycophenolate | 4 (0.36%) | 15 (1.09%) |
| Tacrolimus | 9 (0.81%) | 15 (1.09%) |
| Azathioprine | 59 (5.31%) | 21 (1.52%) |
| Cyclosporine | 2 (0.18%) | 1 (0.07%) |
| Dose of Corticosteroids: |  |  |
| 0 | 921 (82.8%) | 1046 (75.7%) |
| <= 10 mg/d | 155 (13.9%) | 286 (20.7%) |
| >10 mg/d | 36 (3.24%) | 50 (3.62%) |
| Anti-hypertensive drugs | 252 (22.7%) | 432 (31.3%) |
| ACE inhibitors | 160 (14.4%) | 237 (17.1%) |
| ARBs | 96 (8.63%) | 197 (14.3%) |
| Chronic NSAIDs | 202 (18.2%) | 296 (21.4%) |
| CV= cardiovascular. DMARDs= disease modifying antirheumatic drugs. bDMARDs= biologic DMARDs. sDMARDs = synthetic DMARDs. JAK=Janus kinase. IL=interleukin. TNF=tumor necrosis factor. NSAIDs= non-steroid anti-inflammatory drugs. ACE= angiotensin-converting enzyme. ARBs= angiotensin II receptor blockers.  bDMARDs include anti-TNFα, pro-inflammatory ILs antagonists, vedolizumab and T and B lymphocyte antagonists.  sDMARDs include methotrexate, JAK inhibitors, sulfasalazine, mycophenolate, tacrolimus, azathioprine, cyclosporine, chloroquine or hydroxychloroquine, leflunomide and apremilast.  Anti-hypertensive drugs include ACE inhibitors and ARBs. | | |

**Supplementary Table 5. Crude and age-standardized cumulative incidence rates of COVID-19 in our cohort stratified by the exposure to bDMARDs, registered from March 1st to March 29th, 2020.**

|  | Population exposed to bDMARDs | | Population unexposed to bDMARDs | | ALL population | |
| --- | --- | --- | --- | --- | --- | --- |
|  | Crude cumulative incidence rate | Age-standardized  cumulative incidence rate of COVID-19 | Crude cumulative incidence rate | Age-standardized  cumulative incidence rate of COVID-19 | Crude cumulative incidence rate | Age-standardized  cumulative incidence rate of COVID-19 |
| 20-29 | 5 / 76 (6.58%) | 0% | 0 / 0 (0%) | 0% | 5 / 102 (0.7%) | 0.7% |
| 30-39 | 3 / 155 (1.94%) | 0.36% | 3 / 54 (5.56%) | 2.93% | 6 / 209 (2.87%) | 0.53% |
| 40-49 | 12 / 254 (4.72%) | 0.89% | 15 / 149 (10.07%) | 1.89% | 27 / 403 (6.7%) | 1.26% |
| 50-59 | 10 / 256 (3.91%) | 0.63% | 24 / 258 (9.3%) | 1.49% | 34 / 514 (6.61%) | 1.06% |
| 60-69 | 6 / 231 (2.6%) | 0.34% | 23 / 347 (6.63%) | 0.86% | 29 / 578 (5.02%) | 0.65% |
| 70-79 | 3 / 115 (2.61%) | 0.27% | 29 / 356 (8.15%) | 0.86% | 32 / 471 (6.79%) | 0.71% |
| 80-89 | 3 / 22 (13.64%) | 0.97% | 16 / 172 (9.3%) | 0.66% | 19 / 194 (9.79%) | 0.7% |
| 90 + | 0 / 3 (0%) | 0% | 4 / 20 (20%) | 0.38% | 4 / 23 (17.39%) | 0.33% |
| All | 42 / 1112  3.78%  (95%CI 2.66-4.9%) | 3.45%  (95%CI 2.38-4.53%) | 114 / 1382  8.25%  (95%CI 6.8-9.7%) | 6.17%  (95%CI 4.9-7.44%) | 156 / 2494  6.26%  (95%CI 5.3-7.21%) | 5.94%  (95%CI 5.01-6.87%) |
| bDMARDs = biologic disease modifying antirheumatic drugs. | | | | | | |

**Supplementary Table 6. Model 4^A^: Adjusted relative risk (RR)* and 95% confidence interval (95%CI) of COVID-19 according to the presence of several comorbidities, treatments and interactions between treatments, stratified by sex**

|  | **All** | | **Women** | | **Men** | |
| --- | --- | --- | --- | --- | --- | --- |
|  | **N** | **RR (95% CI)** | **N** | **RR (95% CI)** | **N** | **RR (95% CI)** |
| **Clinical characteristics** |  |  |  |  |  |  |
| Women | 1592 | 1.06 (0.76, 1.47) |  |  |  |  |
| Age | 2494 | 1 (0.98, 1.01) | 1592 | 1 (0.99, 1.02) | 902 | 0.98 (0.96, 1) |
| CV disease | 290 | 1.1 (0.71, 1.69) | 179 | 1.22 (0.74, 2.02) | 111 | 0.88 (0.38, 2.04) |
| Diabetes | 302 | 1.73 (1.15, 2.61) | 174 | 1.81 (1.12, 2.93) | 128 | 1.45 (0.66, 3.18) |
| Pulmonary disease | 364 | 1.47 (1.02, 2.11) | 241 | 1.47 (0.95, 2.27) | 123 | 1.33 (0.66, 2.71) |
| Kidney disease | 129 | 1.3 (0.7, 2.42) | 76 | 0.91 (0.4, 2.08) | 53 | 2.18 (0.79, 6.01) |
| Cancer or active treatment | 70 | 1.21 (0.54, 2.7) | 47 | 1.1 (0.41, 2.99) | 23 | 1.47 (0.39, 5.52) |
| **Treatments** |  |  |  |  |  |  |
| Biologic DMARDs1 | 1112 | 0.35 (0.21, 0.58) | 579 | 0.25 (0.11, 0.56) | 533 | 0.49 (0.23, 1.07) |
| Synthetic DMARDs2 | 850 | 0.38 (0.22, 0.67) | 583 | 0.44 (0.23, 0.87) | 267 | 0.37 (0.13, 1.03) |
| Glucocorticoids |  |  |  |  |  |  |
| ≤ 10 mg/day | 441 | 0.96 (0.54, 1.72) | 347 | 0.89 (0.43, 1.83) | 94 | 1.67 (0.61, 4.55) |
| > 10 mg/day | 86 | 1.47 (0.54, 3.95) | 62 | 1.71 (0.61, 4.8) | 24 | NA |
| Anti-hypertensive3 | 684 | 1.18 (0.8, 1.73) | 428 | 1.15 (0.76, 1.75) | 256 | 1.2 (0.48, 3.01) |
| Chronic NSAIDs | 498 | 1.26 (0.84, 1.9) | 345 | 1.2 (0.76, 1.9) | 153 | 1.29 (0.56, 2.99) |
| *Interactions* |  |  |  |  |  |  |
| Biologic + Synthetic DMARDs | 283 | 4.3 (2, 9.25) | 178 | 7.22 (2.51, 20.75) | 105 | 2.06 (0.58, 7.36) |
| Synthetic DMARDs + Glucocorticoids (≤ 10 mg/day) | 284 | 1.17 (0.49, 2.8) | 224 | 0.92 (0.31, 2.75) | 60 | 1.63 (0.37, 7.25) |
| Synthetic DMARDs + Glucocorticoids (> 10 mg/day) | 52 | 1.46 (0.38, 5.62) | 35 | 0.85 (0.18, 4.01) | 17 | NA |
| Biologic DMARDs + Anti-hypertensive | 252 | 0.57 (0.25, 1.34) | 116 | 0.33 (0.07, 1.5) | 136 | 0.71 (0.2, 2.51) |
| Biologic DMARDs +Chronic NSAIDs | 202 | 0.93 (0.4, 2.16) | 125 | 0.72 (0.23, 2.28) | 77 | 1.24 (0.33, 4.61) |
| *Reference categories for clinical characteristics are individuals without that comorbidity. Reference categories for treatments are unexposed individuals.  AModel 4 contains the following explanatory or exposure variables: sex, age, CV disease, pulmonary disease, kidney disease, active cancer or treatment, interaction biologic & synthetic DMARDs, , interaction synthetic DMARDs & glucocorticoids, interaction biologic DMARDs & anti-hypertensive drugs and interaction biologic DMARDs & chronic NSAIDs.  CV= cardiovascular. DMARDs= disease modifying antirheumatic drugs. JAK=Janus kinase. IL=interleukin. TNF=tumor necrosis factor. NSAIDs= non-steroid anti-inflammatory drugs. ACE= angiotensin-converting enzyme. ARBs= angiotensin II receptor blockers. hsCOVID-19= high suspicious COVID-19 symptoms  1Biologic DMARDs include Anti-TNF-α, pro-inflammatory ILs antagonists, vedolilzumab and T and B lymphocyte antagonists.  2Synthetic DMARDs include methotrexate, JAK inhibitors, sulfasalazine, mycophenolate, tacrolimus, azathioprine, cyclosporine, chloroquine or hydroxychloroquine and leflunomide and apremilast.  3Anti-hypertensive drugs include ACE inhibitors and ARBs. | | | | | | |

**Supplementary Table 7. Description the matched dataset for the exposure to anti-TNFα after propensity score matching.**

|  | **Anti-TNFα** | | |
| --- | --- | --- | --- |
| **Sample size** |  | control | treated |
|  | N All | 1726 | 768 |
|  | N matched | 768 | 768 |
|  | N  unmatched | 958 | 0 |
| **hsCOVID-19 symptoms (matched data)** | Yes | 36 | 29 |
|  | No | 732 | 739 |
| **Summary of balance for matched data** | Means Treated | Means Control | Mean Diff |
| Women | 0.51 | 0.60 | -0.10 |
| Age | 51.72 | 57.02 | -5.30 |
| CV disease | 0.07 | 0.10 | -0.03 |
| Diabetes | 0.09 | 0.13 | -0.04 |
| Pulmonary disease | 0.11 | 0.14 | -0.03 |
| Renal disease | 0.03 | 0.05 | -0.01 |
| Cancer | 0.01 | 0.01 | 0.00 |
| Rheumatoid Arthritis | 0.24 | 0.43 | -0.19 |
| Spondylarthritis | 0.67 | 0.38 | 0.29 |
| Juvenile rheumatoid arthritis | 0.01 | 0.00 | 0.00 |
| Dermatological diseases | 0.07 | 0.17 | -0.10 |

**CLINICAL RESEARCH PROTOCOL**

*Observational study to evaluate the potential effects of biological, biosimilar, and targeted synthetic disease-modifying antirheumatic drugs in the appearance of symptoms compatible with COVID-19 infection*

| **Protocol version and date** | Version: 1.0  Date: 04/13/2020 |
| --- | --- |
| **Protocol code** | PreCOVIDMar |
| **Short title or acronym** | PreCOVIDMar |
| **Sponsor** | Barcelona MAR Health Park Consortium (Parc de Salut MAR) Hospital del Mar building  Passeig Marítim 25-29  08003 Barcelona |
| **Research team** | **Principal Investigator**  Dr. Jordi Monfort Faure  Cell Research on Inflammation and Cartilage. Hospital del Mar Medical Research Institute (IMIM). Rheumatology Service, Hospital del Mar  Address: Passeig Marítim 25-29, 08003 Barcelona  Phone: +34 93 248 33 32  E-mail: [JMonfort@parcdesalutmar.cat](mailto:JMonfort@parcdesalutmar.cat)  **Co-Investigator**  Dr. Rafael Maldonado López  Department of Experimental and Health Sciences. Neuropharmacology Research group. Hospital del Mar Medical Research Institute (IMIM). Pompeu Fabra University  Address: Dr. Aiguader, 88 08003 Barcelona  Phone: +34 933160824  E-mail: [rafael.maldonado@upf.edu](mailto:rafael.maldonado@upf.edu)  **Collaborating reasearchers**  Laura Tio Barrera  Cell Research on Inflammation and Cartilage. Hospital del Mar Medical Research Institute (IMIM). Rheumatology Service, Hospital del Mar  Jone Llorente Onaindia  Cell Research on Inflammation and Cartilage. Hospital del Mar Medical Research Institute (IMIM). Rheumatology Service, Hospital del Mar  Luciano Polino  Cell Research on Inflammation and Cartilage. Hospital del Mar Medical Research Institute (IMIM). Rheumatology Service, Hospital del Mar  Natàlia Soldevila Domènech  Integrative pharmacology and systems neuroscience. Hospital del Mar Medical Research Institute (IMIM). |
|  | Alba Gurt Davi  Primary care centre Vila Olímpica. Parc Sanitari Pere Virgili. |

**TABLE OF CONTENTS**

[1. Summary ................................................................................................... 4](#_TOC_250010)

[2. Abbreviations.............................................................................................. 7](#_TOC_250009)

[3. History of versions...................................................................................... 7](#_TOC_250008)

[4. Theoretical framework................................................................................ 8](#_TOC_250007)

5. Objectives................................................................................................... 9

[6. Methodology .............................................................................................. 9](#_TOC_250006)

[7. Study procedures……………..................................................................... 13](#_TOC_250005)

[8. Period of study .......................................................................................... 14](#_TOC_250004)

[9. Statistical methods….................................................................................. 14](#_TOC_250003)

[10. Ethical aspects ......................................................................................... 14](#_TOC_250002)

[11. Confidentiality of data………. ................................................................... 15](#_TOC_250001)

[12. References ................................................................................................ 15](#_TOC_250000)

# SUMMARY

| **Title** | Observational study to evaluate the potential effects of biological, biosimilar,  and targeted synthetic disease-modifying antirheumatic drugs in the appearance of symptoms compatible with COVID-19 infection |
| --- | --- |
| **Study Description** | Observational, retrospective study to compare the cumulative incidence of symptoms compatible with COVID19 infection during the period 1st to 29th March 2020 between patients receiving disease-modifying anti-rheumatic drugs (DMARDs; including biologic gents [bDMARDs], biosimilar agents [bsDMARDs], and targeted synthetic drugs [tsDMARDs) and patients with  other rheumatic diseases not treated with these drugs |
| **Objectives** | **Primary objective**   - To study the cumulative incidence of symptoms compatible with coronavirus infection in patients treated with bDMARDs, bsDMARDs, and tsDMARDs compared to a similar population of patients not treated with DMARDs.   **Secondary objectives**   - To investigate whether there is an immunosuppressive   /immunomodulatory treatment with a protective effect on symptoms compatible with coronavirus infection   - To study the association between ACEi (angiotensin converting enzyme inhibitors) and AIIAs (angiotensin II AT1 receptor antagonists) and the development of symptoms compatible with coronavirus infection - In the case of corticosteroids, to study the possible effect of the dose used - To study the possible differential effects of these drugs depending on the patient’s gender and age - To investigate which comorbidities and treatments or combinations of treatments may be associated with a lower or higher risk of showing symptoms compatible with coronavirus infection or influence the severity of the disease - To investigate the demographic and clinical characteristics of patients with confirmed or strong suspicion of coronavirus infection in order to find associations that can help in the management of the disease and to identify patients with greater or lesser risk of infection |
| **Inclusion and exclusion criteria** | Inclusion criteria for both groups:   - Adults >18 years of age - Referred from any of the primary care centres (PPCs) of the area of influence of the Hospital del Mar - With data in the medical history of the Hospital del Mar available for at least 80% of the values of the variables under study   Study population:   - Patients receiving bDMARDs, bsDMARDs, or tsDMARDs at the time of their inclusion in the study and prescribed for at least three months before inclusion - Patients whose adherence to treatment was good or otherwise not |

|  | recorded in the patient's medical history Control group:   - Patients with rheumatologic diseases not treated with bDMARDs,   bsDMARDs, or tsDMARDs   - Visited during the last 6 months at the Rheumatology service of Hospital del Mar   Exclusion criteria for both groups:   - No access to clinical courses from the family and community medicine specialist - Alive before the 29th March 2020 - Having a negative result in the SARS-VOC-2 detection test |
| --- | --- |
| **Variables** | **Main variables**   - Treatment with bDMARDs, bsDMARDs, or tsDMARDs - Treatment with cMARDs - Treatment with corticosteroids - Consulted for symptoms of COVID-19 infection   **Secondary variables**   - Result issued for the test for SARS-COV-2 detection - Home isolation - Discharged from any CatSalut emergency service - Hospitalisation related with coronavirus infection - Death from confirmed SARS-COV-2 virus infection - Treated with ACEIs - Treated with AIIAs - Gender - Smoking status - Baseline disease (study population) - Other comorbidities |
| **Population and number of subjects** | The medical history of 2,551 patients will be reviewed: 1,701 adult patients treated with bDMARDs, bsDMARDs, or tsDMARDs, and 850 similar  patients not treated with these drugs |
| **Participating centres** | Hospital del Mar, Parc de Salut Mar  Primary care centres in the area of influence of Hospital del Mar   - *PCC Vila Olímpica* - *PCC Barceloneta* - *PCC Besòs* - *PCC La Mina* - *PCC El Clot* - *PCC CAP Sant Martí 2* - *PCC Drassanes* - *PCC Raval Nord* - *PCC Poble Nou* - *PCC Ramón Turró* - *PCC Gòtic* - *PCC Casc Antic* - *PCC La Pau* |
| **Overall duration of the study** | Maximum 12 days from 16th April |

# ABBREVIATIONS

| AIIAs | Angiotensin II AT1 receptor antagonists |
| --- | --- |
| ACEI | Angiotensin converting enzyme inhibitors |
| bDMARDs | Biologic disease-modifying anti-rheumatic drugs |
| bsDMARDs | Biosimilar disease-modifying anti-rheumatic drugs |
| cDMARD | Conventional disease-modifying anti-rheumatic drugs |
| COVID-19 | Coronavirus infection |
| COPD | Chronic obstructive pulmonary disease |
| CRF | Case report form |
| DM | Diabetes Mellitus |
| DMARDs | Disease-modifying anti-rheumatic drugs |
| GCP | Good clinical practice |
| HT | Arterial hypertension |
| ICH | International Conference on Harmonization |
| IL | Interleukin |
| OSAS | Obstructive sleep apnoea syndrome |
| PCC | Primary care centre |
| SAD | Systemic autoimmune disease |
| SARS-COV-2 | Severe acute respiratory syndrome due to Coronavirus 2 |
| SARS | Severe acute respiratory syndrome |
| TNF | Tumour necrosis factor |
| tsDMARD | Targeted synthetic disease-modifying anti-rheumatic drugs |

# HISTORY OF VERSIONS

| **Version** | **Date** | **Description of the change** | **Brief justification** |
| --- | --- | --- | --- |
| 1.0 | 13/04/2020 | NA | NA |

# THEORETICAL FRAMEWORK

**Background and rationale of the proposed study**

Since December 2019, cases of coronavirus infection (COVID-19) responsible for severe acute respiratory syndromes (SARS-CoV-2) have been reported in China. Coronaviruses are important pathogens for humans that appear to be of animal origin like other zoonoses. COVID-19 infection causes a respiratory infection with a clinical presentation that ranges between mild to serious (1, 2).

Severe forms develop in approximately 15% of the patients who have been diagnosed with COVID-19 infection (3). However, it is possible that, at the present time, this is not accurate if we consider the possibility of underrecognized infections for the calculations. Among severe forms, pneumonia is one of the most frequent presentation that can also evolve into severe acute respiratory syndromes (SARS) (4). The pathophysiological mechanisms of these pneumonic conditions seem related to an abnormal and massive activation of the inflammatory response of the organism itself. The most severe forms of COVID-19 infection require the patient’s hospitalization and it is associated with a mortality that currently ranges between 7.2% in Italy (5, 6) and 0.9% in South Korea (7).

Evidence suggests that the hyperactivation of the immune response is of paramount importance in COVID-19 infection. The natural history of the infection has typical clinical and analytical characteristics. A rapid clinical deterioration has been described in the literature between 7 and 15 days after the onset of symptoms (8, 9). It is characterized by a worsening of the respiratory function in which infiltrates of monocytic and macrophage cells are observed, as well as multiorgan failures secondary to hypercoagulability and vasculitis processes (4). This systemic inflammation state of has been compared to that also occurring in two systemic autoimmune diseases (SAD): macrophage activation syndrome and antiphospholipid syndrome.

This pathologically exacerbated immune response can, in severe cases, trigger a cytokine storm. The cytokine storm is an abnormal and excessive inflammatory response mediated by pro-inflammatory cytokines that usually originates from multiple causes, among which are infectious processes and autoimmune diseases (4). At the clinical level, it manifests as elevated inflammatory parameters in peripheral blood, multi-organ failure, and systemic inflammation. In the case of COVID-19 infection, it is presumed that the high viral replication level in the lungs, pulmonary inflammatory infiltrates, and the secondary cytokine storm, are responsible for both the acute serious respiratory syndrome (SARS-COV-2) and the multiorgan failure that can lead to the death of some patients (10, 11).

The increasing knowledge on the pathophysiology of this disease points to different molecules that are part of the main inflammatory pathways in its etiopathogenesis. We currently have specific treatments used in the routine management of various SADs that inhibit some of these pathways. The clearest example is interleukin-6, which contribution to the development of severe forms of the disease is not only known, but its inhibition is one of the main available resources in patients with torpid evolution (4). Other examples of involved molecules which inhibition represents a potential therapeutic alternative are tumour necrosis factor (TNF)-alpha and interleukin 1, which respective inhibitory drugs are anti-TNF-alpha and anakinra / canakinumab / rilonacept (4).

Another sign of the importance of this immune hyperactivation is the efficacy of hydroxychloroquine in the treatment of the disease (12, 13). Of note, hydroxychloroquine is the mainstay immunomodulatory therapy for certain SADs such as systemic lupus erythematosus.

Given the progressive increase in the pandemic in relation to the available health resources, it is critical to find new effective treatments, especially when everything indicates that a rapid shortage of the stock of some of the existing treatments is likely. In these circumstances, any drug with a potential protective effect against the exacerbation of symptoms would be a valuable tool to prevent serious manifestations of the disease, future deaths, and ultimately the collapse of the health system.

**Hypothesis**

- bDMARDs, bsDMARDs, or tsDMARDs decrease the risk of developing symptoms compatible with coronavirus

1. **OBJETIVES Primary objective**
   - To study the cumulative incidence of symptoms compatible with coronavirus infection in patients treated with

bDMARDs, bsDMARDs, and tsDMARDs compared with a similar population of patients not treated with DMARDs.

**Secondary objectives**

- To investigate whether there is an immunosuppressive /immunomodulatory treatment with a protective effect on symptoms compatible with coronavirus infection
- To study the association between ACEi (angiotensin converting enzyme inhibitors) and AIIAs (angiotensin II AT1 receptor antagonists) and the development of symptoms compatible with coronavirus infection
- In the case of corticosteroids, to study the possible effect of the dose used
- To study the possible differential effects of these drugs depending on the patient’s gender and age
- To investigate which comorbidities and treatments or combinations of treatments may be associated with a lower or higher risk of showing symptoms compatible with coronavirus infection or influence the severity of the disease
- To investigate the demographic and clinical characteristics of patients with confirmed or strong suspicion of coronavirus infection in order to find associations that can help in the management of the disease and to identify patients with greater or lesser risk of infection

# METHODOLOGY

- 1. **Study design**

Observational, retrospective study to compare the cumulative incidence of symptoms compatible with COVID19 infection during the period 1st to 29th March 2020 between patients receiving bDMARDs, bsDMARDs, and tsDMARDs and patients with other rheumatic diseases not treated with these drugs

- 1. **Study population, inclusion / exclusion criteria, and criteria for subject’s withdrawal**

Inclusion criteria for both groups:

- - - Adults >18 years of age
    - Referred from any of the primary care centres (PPCs) of the area of influence of the Hospital del Mar
    - With data in the medical history of the Hospital del Mar available for at least 80% of the values of the variables under study

Inclusion criteria for the study population:

- - - Patients receiving bDMARDs, bsDMARDs, or tsDMARDs at the time of their inclusion in the study and prescribed for at least three months
    - Patients whose adherence to treatment was good or otherwise not recorded in the patient's medical history Inclusion criteria for the control group:
    - Patients with rheumatologic diseases not treated with bDMARDs, bsDMARDs, or tsDMARDs
    - Visited during the last 6 months at the Rheumatology service of Hospital del Mar Exclusion criteria for both groups:
    - No access to clinical courses from the family and community medicine specialist
    - Alive before the 29th March 2020
    - Having a negative result in the SARS-VOC-2 detection test
  1. **Definition of the study variables**
     1. **Main variables**
        - Treatment with bDMARDs, bsDMARDs, or tsDMARDs. The patient will be considered to be in treatment when this is stated in his/her updated hospital prescription and regularly picks the treatment from the pharmacy
        - Treatment with bDMARDs and bsDMARDs:
          - Anti-TNF (Etanercept, Infliximab, Adalimumab, Golimumab, and Certolizumab)
          - Anti-IL6 (Sarilumab and Tocilizumab)
          - Anti-other ILs (Ustekinumab, Brodalumab, Guselkumab, Secukinumab, Ixeizumab, and Anakinra)
          - Anti-T lymphocytes (Abatacept)
          - Anti-B lymphocytes (Rituximab and Belimumab)
          - Others: Vedolizumab
        - Treatment with tsDMARDs (Tofacitinib, Baricitinib, and Upadacitinib)
        - Treatment with cDMARDs. The patient will be considered to be in treatment when this is stated in his/her updated electronic prescription and his/her doctor does not record in the clinical course that the patient does not take them.
          - Leflunomide
          - Sulfasalazine
          - Methotrexate
          - Tacrolimus
          - Azathioprine
          - Cyclosporine
          - Mycophenolate / mycophenolic acid
          - Hydroxychloroquine / Chloroquine
          - Apremilast
        - Treatment with corticosteroids. The patient will be considered to be in treatment when it is stated in his/her updated electronic prescription and his doctor does not record in the clinical course that the patient does not take them. The different corticosteroids will be adjusted to the equivalent dose of prednisone.
        - Consultation for symptoms of COVID-19 infection. Consultations from the 1st to the 29th March 2020 in patients with the following symptoms will be considered as positive:
          - Body temperature above 37ºC plus asthenia and / or dry cough. In the absence of asthenia and / or dry cough, those with two or more of the following symptoms: anosmia, ageusia, rhinorrhoea, diarrhoea, pharyngitis, or odynophagia and arthromyalgia.
     2. **Secondary variables**
- Positive result of the SARS-COV-2 detection test by any of the accredited reference laboratories in Catalonia between the 1st and the 29th March 2020.
- Home isolation indicated by a doctor and related to symptoms compatible with COVID-19 infection manifested between the 1st and the 29th March 2020.
- Discharge from any CatSalut emergency service that did not require hospitalisation between the 1st and the 29th March 2020.
- Hospitalisation related with coronavirus infection in any hospital accessed via the HC3 between the 1st and the 29th March 2020.
- Death from confirmed SARS-COV-2 virus infection when certified by a doctor.
- Treatment with ACEIs if specified in the updated electronic prescription
- Treatment with AIIAs if specified in the updated electronic prescription
- Gender
  - Man
  - Woman
- Smoking: it will be recorded whether the subject is or not a smoker, the years that he/her has been smoking or not smoking (in the case of ex-smokers) and the number of cigarettes / day
- Base diseases in the study population: SAD diagnosed by an accredited doctor and stated in the patient's medical history.
  - Rheumatoid arthritis
  - Psoriatic arthritis
  - Spondyloarthritis
  - Ulcerative colitis
  - Crohn's disease
  - Psoriasis
  - Behçet disease
  - Lupus
  - Pyoderma gangrenosum
  - Still's disease
  - Amyloidosis
  - Polyarteritis nodosa
  - Eosinophilic fasciitis
  - Juvenile idiopathic arthritis
  - Autoinflammatory disease
  - Uveitis
  - Hidradenitis
  - Sjögren syndrome
- Other comorbidities. The clinical diagnoses from the hospital clinical history, from the PCC, and from all available reports will be reviewed. The following comorbidities of interest will be recorded:
  - Diagnosed with hypertension (HT) in medical treatment
  - Lung disease (asthma, chronic obstructive pulmonary disease [COPD], obstructive sleep apnoea syndrome [OSAS], interstitial lung disease, cystic fibrosis, and others)
  - Need for home oxygen
  - Diagnosed with cardiovascular disease
  - Diagnosed with chronic kidney disease
  - Diabetes mellitus (DM): we will register whether the patient has been diagnosed with DM and if he/she is being treated with oral antidiabetics or insulin
  - Transplant: The presence of transplanted organs and / or tissues will be recorded
  - Cancer and / or active treatment until 29th March. In those patients with a history of cancer that show cure criteria documented by an oncologist, cancer will not be considered as a comorbidity.

No criteria for withdrawal of subjects will be considered; the data of all patients who meet all the inclusion criteria will be analysed

# STUDY PROCEDURES

To obtain study the population, a list of all patients being treated with bDMARDs, bsDMARDs, or tsDMARDs, will be requested to the PCCs of the area of reference of the Hospital del Mar and the CatSalut.

To obtain the control group, the records from the 1st September 2019 to the 29th February 2020 of the monographic medical dispensaries of the Rheumatology service of Hospital del Mar will be reviewed.

A database or case report form (CRF) will be designed with the Access® software, and will include all the variables under study. Five researchers will review the clinical history of the potential patients to be included in both cohorts. Only those who meet all the inclusion criteria and none of the exclusion criteria will be included for further analysis. In accordance with the General Data Protection Regulation 2016/679 and the Organic Law 3/2018 of the 5th December on Protection of Personal Data and Guarantee of Digital Rights, a unique code will be assigned to each patient and will not include any personal data that could make the subject of the study identifiable. The patient identification code will consist of a number assigned to each investigator, followed by a number assigned to patients in order of entry into the database. As an example, patient number 234 included by researcher number 3 will be assigned the number 3-234.

All the data that include the described variables, available and updated as of 29th March, will be recorded, so that no new treatments, diagnoses or symptoms will be recorded from that date onwards.

This observational study does not include other additional tests or procedures that may pose additional risks for the patient.

# PERIOD OF STUDY

The collection of data in the electronic CRF will start on 16th April 2020, and it is estimated that they will be ready for publication before the 1st May 2020.

# STATISTICAL METHODS

- 1. **Calculation and / or justification of the sample size**

Accepting an alpha risk of 0.05 and a beta risk of 0.2 in a bilateral contrast, 1,701 subjects are required in the exposed group and 850 in unexposed group to detect a minimum relative risk of 1.3 and if the rate of patients in the unexposed group is 0.15. It has been estimated the follow-up loss rate will be 0%. For these calculations, the POISSON approximation was used.

- 1. **Statistical analyses**

An exhaustive descriptive analysis of all the variables of interest will be carried out. The analyses will include measures of central tendency and dispersion for numerical variables, as well as absolute and relative frequencies for categorical variables. The number of missing data will be reported in each case. To evaluate the associations between the different treatments and the appearance of symptoms of COVID-19, Poisson regression models with robust estimation of variance will be used to estimate incidence rates and 95% confidence intervals, excluding those individuals with negative confirmation in the test of the SARS-COV-2.

# ETHICAL ASPECTS

The study will be conducted in accordance with the ethical principles derived from the Declaration of Helsinki (Fortaleza, Brazil, October 2013). In addition, the study will be conducted in accordance with protocol, the International Conference on Harmonization (ICH) guideline for Good Clinical Practice (GCP), and the regulatory requirements for the participating institutions.

The study will be carried out according to a protocol reviewed by qualified personnel from an Ethics Committee. The benefits of the study are considered to be in proportion to the risks and the rights and well-being of the subjects will be respected.

Due to the nature of the study (all the data will be completely anonymous), the importance of immediate results, and their implication for the treatment of the SARS-COV-2 pandemic, it is not planned to obtain informed consent from the participants.

# CONFIDENTIALITY OF DATA

This study will be conducted in accordance with the General Data Protection Regulation (RGPD) No 2016/679 of the European Parliament and of the Council of 27 April 2016 and the Organic Law 3/2018, of 5 December, of Protection of Personal Data and Guarantee of Digital Rights. A unique code will be assigned to each patient and no personal data or data that could make the study subject identifiable will be included (pseudo-anonymised data). The patient identification code will consist of a number assigned to each investigator, followed by a number assigned to patients in order of entry into the database.

The 5 researchers who will have access to the clinical history of the patients to be included in the study are hospital doctors who have personal codes for their routine clinical practice and will ensure the protection of all personal data. No other research staff will have access the patient's history or will be able to consult any data that is not included in the CRF, always appropriately and completely pseudo-anonymised.

The study data will be verifiable against the source data, all the original records, laboratory reports, and subject records will remain in the corresponding hospital medical documentation and on the HC3 portal. The confidentiality of the data and the identity of the patients will be maintained during the study and after its completion. Only the Principal Investigator and authorized study staff will have access to these confidential records. All original data will be in the IMASIS and the HC3 shared history. The Principal Investigator is responsible for ensuring that no paper copy will be kept.

No data used in the analysis and subsequent dissemination of the study results will contain any identifiable reference regarding the names of the patients.

Once the study is completed, the results will be communicated to the competent authorities in a convenient manner and in accordance with the local legislation.

It is anticipated that the results will be published in indexed scientific journals and the study will be registered in ClinicalTrials.gov.

# References

1. Cai Q, Yang M, Liu D, Chen J, Shu D, Xia J, et al. Experimental Treatment with Favipiravir for COVID-19: An Open-Label Control Study. *Engineering* 2020; doi.org/10.1016/j.eng.2020.03.
2. Chan JF, Yuan S, Kok KH, To KK, Chu H, Yang J, et al. A familial cluster of pneumonia associated with the 2019 novel coronavirus indicating person-to-person transmission: a study of a family cluster*. Lancet* 2020;**395**(10223):514- 523.
3. Wu Z, McGoogan JM. Characteristics of and Important Lessons from the Coronavirus Disease 2019 (COVID-19) Outbreak in China: Summary of a Report of 72 314 Cases from the Chinese Center for Disease Control and Prevention. *JAMA 2020*; **323**(13):1239-1242.
4. Zhang W, Zhao Y, Zhang F, Wang Q, Li T, Liu Z, et al. The use of anti-inflammatory drugs in the treatment of people with severe coronavirus disease 2019 (COVID-19): The experience of clinical immunologists from China. *Clin Immunol*. 2020;**214**(March):108393.
5. Grasselli G, Pesenti A, Cecconi M. Critical Care Utilization for the COVID-19 Outbreak in Lombardy, Italy: Early Experience and Forecast During an Emergency Response. *JAMA* 2020; Mar 13 doi: 10.1001/jama.2020.4031.
6. Onder G, Rezza G, Brusaferro S. Case-Fatality Rate and Characteristics of Patients Dying in Relation to COVID-19 in Italy. *JAMA* 2020; Mar 23. doi:10.1001/jama.2020.4683
7. KCDC. Updates on COVID-19 in Korea. March 14, 2020.

<https://www.cdc.go.kr/board/board.es?mid=a30402000000&bid=0030>(accessed Apr 18, 2020).

1. Li Q, Guan X, Wu P, et al. Early Transmission Dynamics in Wuhan, China, of Novel Coronavirus-Infected Pneumonia. *N Engl J Med* 2020;382(13):1199–1207. doi:10.1056/NEJMoa2001316
2. 1. Guan W, Ni Z, Hu Y, Liang W, Ou C, He J, et al. Clinical Characteristics of Coronavirus Disease 2019 in China. *N Engl J Med* 2020;1–13.
3. Channappanavar R, Perlman S. Pathogenic human coronavirus infections: causes and consequences of cytokine storm and immunopathology. *Semin Immunopathol* 2017 Jul;**39**(5):529-539.
4. Chousterman BG, Swirski FK, Weber GF. Cytokine storm and sepsis disease pathogenesis. *Semin Immunopathol*

2017;**39**(5):517-528.

1. Colson P, Rolain JM, Raoult D. Chloroquine for the 2019 novel coronavirus SARS-CoV-2. *Int J Antimicrob Agents*

2020;**55**(3):105923.

1. Cortegiani A, Ingoglia G, Ippolito M, Giarratano A, Einav S. A systematic review on the efficacy and safety of chloroquine for the treatment of COVID-19. *J Crit Care* 2020: S0883-9441(20)30390-7. doi: 10.1016/j.jcrc.2020.03.005.


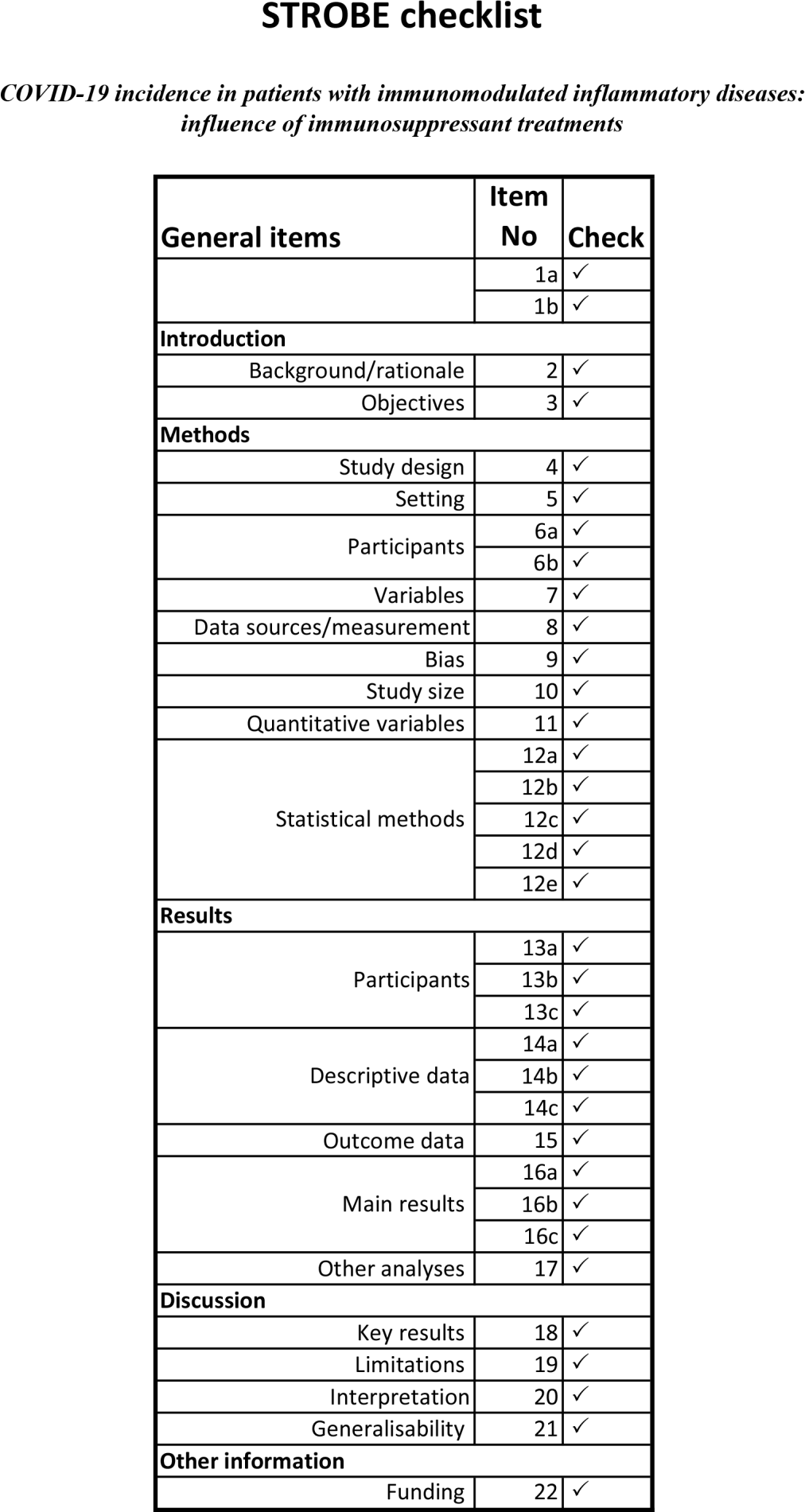

Supplement: Supplementary file 1 [file datasheet1.docx]
